# Anticoagulant drugs for patients with atrial fibrillation on dialysis: a systematic analysis and network meta-analysis

**DOI:** 10.3389/fphar.2023.1320939

**Published:** 2023-12-15

**Authors:** Xian-Feng Shen, Chao Zhang, Jun Hu, Tao Zhang, Bin Ma

**Affiliations:** ^1^ Evidence-Based Medicine Center, School of Basic Medical Sciences, Lanzhou University, Lanzhou, Gansu, China; ^2^ Department of General Surgery, Taihe Hospital, Hubei University of Medicine, Shiyan, Hubei, China; ^3^ Center for Evidence-Based Medicine and Clinical Research, Taihe Hospital, Hubei University of Medicine, Shiyan, Hubei, China; ^4^ Department of Neurosurgery, Taihe Hospital, Hubei University of Medicine, Shiyan, Hubei, China

**Keywords:** end-stage renal disease, dialysis, atrial fibrillation, direct oral anticoagulants, warfarin, network meta-analysis

## Abstract

**Objective:** A lack of clarity persists regarding the efficacy and risks associated with direct oral anticoagulants (DOACs) in end-stage renal disease (ESRD) patients with atrial fibrillation (AF) undergoing dialysis, primarily due to limited retrospective studies. Therefore, the objective of this study was to evaluate the existing data and propose a practical protocol for the clinical utilization of DOACs in ESRD patients with AF undergoing dialysis.

**Methods:** PubMed, EMBASE, and the Cochrane Central Register of Controlled Trials were searched for clinical studies evaluating DOACs in ESRD patients with AF on dialysis published up to 2 February 2023. DOACs included warfarin, dabigatran, apixaban, edoxaban, and rivaroxaban. The outcomes were mortality, ischemic stroke, hemorrhagic stroke, any stroke, gastrointestinal bleeding, major bleeding, intracranial bleeding, and minor bleeding.

**Results:** Compared with placebo, apixaban (HR = 0.97, 95% CI: 0.88–1.07), rivaroxaban (HR = 0.91, 95% CI: 0.76–1.10), and warfarin (HR = 0.96, 95% CI: 0.90–1.01) did not reduce mortality. Regarding direct comparisons of mortality, the comparisons of warfarin vs. apixaban (HR = 0.99, 95% CI: 0.92–1.06), placebo vs. warfarin (HR = 1.04, 95% CI: 0.99–1.11), and rivaroxaban vs. warfarin (HR = 0.96, 95% CI: 0.80–1.14) did not significantly reduce mortality. Based on the surface under the cumulative ranking curve, rivaroxaban (75.53%), warfarin (62.14%), and apixaban (45.6%) were the most effective interventions for managing mortality, and placebo (16.74%) was the worst.

**Conclusion:** In conclusion, rivaroxaban demonstrated efficacy in reducing mortality and the incidence of ischemic stroke, gastrointestinal bleeding, and intracranial hemorrhage. Dabigatran is recommended for the prevention of hemorrhagic stroke. However, caution should be exercised due to the risk of major bleeding. Warfarin can effectively reduce minor bleeding but does not offer significant protection against gastrointestinal or intracranial bleeding. Apixaban was not recommended for mortality reduction or for preventing ischemic or hemorrhagic strokes. Further research will be necessary to establish specific clinical protocols.

## 1 Introduction

Atrial fibrillation (AF) occurred in more than 10% of end-stage renal disease (ESRD) patients undergoing dialysis (Hijazi et al., 2016). In patients with ESRD receiving dialysis, the coexistence of AF substantially augmented the susceptibility to thrombosis owing to perturbations in atrial contractility; diminished atrial blood perfusion, progression of atrial fibrosis, and impairment and dysfunction of the endothelium; and upregulated the expression of tissue factor, leading to enhanced platelet aggregation and augmented fibrinolysis (Proietti et al., 2018). Anticoagulation was associated with a lower incidence of ischemic stroke, preventing thrombosis and reducing the likelihood of death in patients (Ding et al., 2021). Therefore, the imperative necessity for implementing anticoagulation therapy in patients with AF on dialysis was emphasized (Bonde et al., 2014).

The anticoagulant warfarin impeded the synthesis of coagulation factors and mitigated the risk of thrombosis by inhibiting vitamin K epoxide reductase (Mantha and Ansell, 2012). Multiple studies demonstrated the efficacy of warfarin in preventing ischemic stroke in ESRD patients with AF undergoing dialysis, leading to favorable prognoses (Benz and Eikelboom, 2022; See et al., 2021; Yoon et al., 2017; Ntaios et al., 2017). However, it is crucial to acknowledge that warfarin carries an inherent risk of increasing bleeding tendencies in patients (Baker et al., 2023). Therefore, for individuals with AF on dialysis who were more susceptible to bleeding events, there is a pressing need for the development of safer and more effective anticoagulant medications (Potpara et al., 2012). In recent years, direct oral anticoagulants (DOACs) have been extensively used in anticoagulant therapy. The mechanism of action of DOACs was through direct action on certain clotting factors, mainly the thrombin inhibitor dabigatran and factor Xa inhibitors, including rivaroxaban, apixaban, and edoxaban. DOACs directly acted on coagulation factors, simplified the process of anticoagulation therapy, and had less impact on the clotting pathway, so there was no need for routine monitoring of INR, and the risk of bleeding was relatively low (Franchini et al., 2016). DOACs were currently recommended for the prevention of stroke and systemic thromboembolism in patients with non-renal impaired AF, and these drugs were superior to warfarin in reducing bleeding (Edwina et al., 2023). However, all DOACs were primarily eliminated via renal excretion, with apixaban having a renal clearance of 27% and dabigatran reaching up to 80% (Stamellou and Floege, 2018). Consequently, patients with severe renal impairment (e.g., serum creatinine clearance <25–30 mL/min) or ESRD had been systematically excluded from clinical trials involving DOACs (Pokorney et al., 2020). However, in clinical practice, an increasing number of patients with ESRD and AF were opting for DOACs as an alternative to warfarin therapy after experiencing treatment failure (Lip et al., 2017).

Despite the wealth of evidence supporting anticoagulation used in patients with chronic kidney disease (CKD), there remained a lack of clarity regarding the efficacy and risks associated with DOACs in ESRD patients with AF undergoing dialysis, primarily due to limited retrospective studies lacking network meta-analysis (NMA). Therefore, the objective of this study was to evaluate the existing data and propose a practical protocol for the clinical utilization of DOACs in ESRD patients with AF undergoing dialysis.

## 2 Methods

### 2.1 Literature search

Three databases, including PubMed, EMBASE, and the Cochrane Library, were systematically and comprehensively searched to retrieve relevant literature and references published before 02 February 2023. The MeSH terms employed in this study encompassed “renal dialysis,” “hemodialysis,” “chronic kidney disease,” “end-stage renal disease,” “dialysis,” and “atrial fibrillation,” along with the more specific terms of “anticoagulants,” “oral anticoagulants,” “NOACs,” “dabigatran,” “apixaban,” “rivaroxaban,” “edoxaban,” and “warfarin.” The detailed search strategies are deposited in **Supplementary Method 1**.

### 2.2 Inclusion and exclusion criteria

The following inclusion criteria were used (Hijazi et al., 2016): participants: adults diagnosed with atrial fibrillation on dialysis (Proietti et al., 2018); interventions: anticoagulant drugs, including DOACs (apixaban, dabigatran, rivaroxaban, and edoxaban) and warfarin (Ding et al., 2021); comparison: none of the anticoagulant drugs (placebo) or other anticoagulant drugs (Bonde et al., 2014); outcomes: mortality, ischemic stroke, hemorrhagic stroke, any stroke, gastrointestinal hemorrhage, major bleeding, intracranial bleeding, and minor bleeding (Mantha and Ansell, 2012); and study design: randomized controlled trials (RCTs) and cohort studies.

The exclusion criteria were as follows (Hijazi et al., 2016): mixed-population study, patients with renal failure but not on dialysis, reported cardiovascular disease (e.g., coronary artery disease, moderate or severe aortic or mitral stenosis, and active endocarditis), traumatic brain injury, and other non-psychological conditions that may have a greater impact on the patient’s mental state (Proietti et al., 2018); no available data in the original research or the data could not be converted (Ding et al., 2021); prevention of relapse trials (Bonde et al., 2014); cross-linking experiments; and duplicate studies (Mantha and Ansell, 2012).

### 2.3 Data extraction

Data extraction was performed by two independent authors, with the extracted data proofread by a final investigator. If relevant data were not reported in an article, the most recent data were calculated based on the related data reported in the original articles.

### 2.4 Quality assessment

In the quality appraisal of the RCTs, five aspects, including the randomization process, deviations from the intended intervention, missing outcome data, measurement of the outcome, and selection of the reported result, based on a revised tool for assessing the risk of bias in randomized trials from the Cochrane Handbook (RoB-2) (Sterne et al., 2019), were employed.

For the non-randomized trials, the risk of bias in non-randomized studies of interventions (ROBINS-I) tool (Sterne et al., 2016) was employed. The selected items included confounding bias, subject selection bias, intervention classification bias, bias in deviation from established interventions, missing data bias, endpoint measurement bias, and selective reporting bias. Responses to each question were selected from “Yes,” “Probably Yes,” “No,” “Probably No,” “No Information,” and “Not Applicable.” Any disagreement between the two reviewers was resolved by a third reviewer.

### 2.5 Statistical analysis

All outcomes were denoted as hazard ratios (HRs) with corresponding 95% confidence intervals (CIs) using the generated network meta-analysis models. The chi-square test was used to assess heterogeneity, with the level of significance set to *p* < 0.1. I^2^ values > 40% were interpreted as indicating significant heterogeneity; in such circumstances, a random-effects model was used to conduct meta-analysis. On the other hand, for I^2^ values ≤ 40%, a fixed-effect model was used instead. The back-calculation method was employed to test the consistency of all outcomes, using separate indirect and direct evidence. The surface under the cumulative ranking curve (SUCRA) for summarizing probabilities was used to provide summary and pooled statistics for the cumulative ranking. Studies with a mean heart failure rate of less than 20% were excluded in the sensitivity analysis. All statistical analyses were performed using the GeMTC package (versions 1.0–2) of R version 4.2.2 (Vienna, Austria).

## 3 Results

### 3.1 Study selection

**FIGURE 1:**
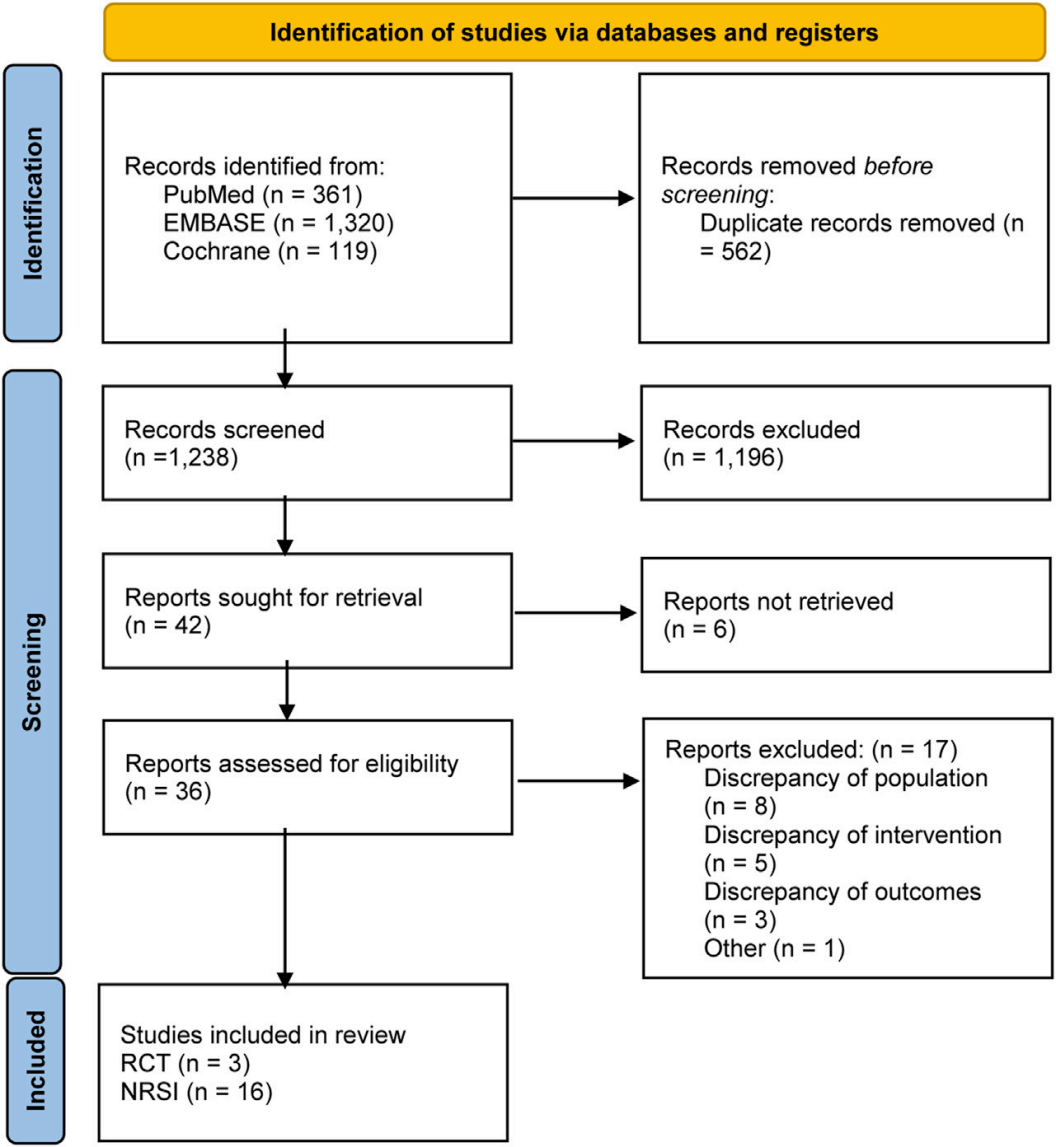
Literature screening for inclusion of studies.

Figure 1 shows the specific screening process. A total of 1,800 articles were retrieved for initial screening, and 562 duplicate articles were first excluded. After the reading of titles and abstracts, 1,238 articles were removed. The articles were comprehensively reviewed, and 17 articles that met the exclusion criteria for this study were removed. A total of 19 studies (Chan et al., 2009; Winkelmayer et al., 2011; Shah et al., 2014; Wakasugi et al., 2014; Chan et al., 2015; Genovesi et al., 2015; Shen et al., 2015; Yodogawa et al., 2016; Kai et al., 2017; Yoon et al., 2017; Siontis et al., 2018; Tan et al., 2019; Mavrakanas et al., 2020; De Vriese et al., 2021; Lin et al., 2021; Pokorney et al., 2022; Sy et al., 2022; Wetmore et al., 2022; Reinecke et al., 2023) were included in this study, comprising three randomized controlled trials (De Vriese et al., 2021; Pokorney et al., 2022; Reinecke et al., 2023) and observational studies.

### 3.2 Characteristics of included studies

A total of 103,684 subjects were included in the analysis, as detailed in Table 1. Most were over 60 years of age, and most were men. The sample size ranged from 60 (29) to 25,523 (22) participants, and mean follow-up periods were 1 (35) to 4 (25) years. Eight studies (Kai et al., 2017; Siontis et al., 2018; De Vriese et al., 2021; Lin et al., 2021; Pokorney et al., 2022; Sy et al., 2022; Wetmore et al., 2022; Reinecke et al., 2023) used CHA_2_DS_2_–VASc scores, and three studies (Chan et al., 2009; Chan et al., 2015; Yodogawa et al., 2016) used the CHADS_2_ score to score subjects. History of stroke and embolism was present in subjects in eight studies (Shah et al., 2014; Chan et al., 2015; Genovesi et al., 2015; Yodogawa et al., 2016; Kai et al., 2017; Tan et al., 2019; De Vriese et al., 2021). Atrial fibrillation occurred before dialysis in three studies consisting of 11,705 patients (Chan et al., 2009; Wakasugi et al., 2014; Sy et al., 2022) and occurred after dialysis in ten studies comprising 59,204 patients (Winkelmayer et al., 2011; Shah et al., 2014; Chan et al., 2015; Genovesi et al., 2015; Shen et al., 2015; Yodogawa et al., 2016; Kai et al., 2017; Siontis et al., 2018; Tan et al., 2019; Mavrakanas et al., 2020). Heart failure, hypertension, and diabetes were present in approximately 70% of the subjects in most studies. The intervention used in most studies was warfarin (Chan et al., 2009; Winkelmayer et al., 2011; Shah et al., 2014; Wakasugi et al., 2014; Genovesi et al., 2015; Shen et al., 2015; Yodogawa et al., 2016; Kai et al., 2017; Yoon et al., 2017; Tan et al., 2019; Sy et al., 2022), followed by rivaroxaban (Chan et al., 2015; De Vriese et al., 2021; Lin et al., 2021). The doses of rivaroxaban ranged from 10 mg daily (De Vriese et al., 2021) to 20 mg daily (Chan et al., 2015). The apixaban doses ranged from 5 mg daily to 10 mg daily (Siontis et al., 2018; Mavrakanas et al., 2020; Pokorney et al., 2022; Wetmore et al., 2022; Reinecke et al., 2023). Patients on dabigatran were treated with 150 mg daily (84.7%) and with the usual dose of 300 mg daily (15.3%) (Chan et al., 2015). No relevant literature regarding edoxaban treatment in patients with AF on dialysis was included.

**TABLE 1:** Basic information of all including studies.

Study	Year	Location	Sample size	Age	Female (%)	CHA_2_DS_2_–VASc score	CHADS_2_ score	Prior stroke or embolization	Heart failure (%)	Hypertension (%)	Diabetes mellitus (%)	Anticoagulant 1	Anticoagulant 2	Follow-up period (year)
Chan	2009	US	1,671	72.6 (0.4)/71.3 (0.6)	42.2/45.6	NA	2.75 (0.05)/2.58 (0.06)	14.4 (2)/11.9 (1)	58.3 (2)/52.9 (2)	79.7 (2)/79.8 (2)	40.6 (1)/34.4 (2)	Warfarin	None	1.6
Chan	2015	US	8,589	68.4/66.9/70.6	40.8/39.5/38.8	NA	2.3 (1.0)/2.2 (1.0)/2.4 (1.0)	NA	14.6/14.1/20.8	86.9/84.9/88.5	70.4/67.8/67.9	Dabigatran 150 mg BID (15.3%) and 75 mg BID (84.7%); rivaroxaban 20 mg QD (32.1%) and 15 mg QD (67.8%)	Warfarin	2
Genovesi	2015	Italy	290	NA	35.8/43.6	NA	NA	27.6/40.4	43.3/36.5	76.1/85.3	29.1/33.3	Warfarin	None	2
Kai	2017	US	1,776	68.9 ± 11.4/68.9 ± 12.4	38.4/36.6	5.2 ± 1.7/5.2 ± 1.8	NA	24/24.1	73.4/75.5	99.2/99.3	79.5/78.4	Warfarin	None	2.1
Lin	2021	Taiwan	3,358	69 ± 11/69 ± 12	57/51	3.8 ± 1.5/3.7 ± 1.6	NA	NA	43/37	78/78	51/50	Rivaroxaban 20 mg QD (10.4%), 15 mg QD (38.7%), and 10 mg QD (50.8%)	Warfarin	1.59
Mavrakanas	2020	US	2,082	68 ± 11/68 ± 13	46/47	NA	NA	34/36	76/76	100/100	80/80	Apixaban 2.5 mg BID (49.3%) and 5 mg BID (39.7%)	None	NA
Pokorney	2022	US	154	69.0(61.0, 76.0)/68.0(60.5–72.5)	41.5/30.6	4.0(3.0, 5.0)/4.0(3.0, 5.0)	NA	NA	52.4/56.9	96.3/93.1	51.2/65.3	Apixaban 2.5 mg BID and 5 mg BID (weight ≤60 kg and/or age ≥80 years)	Warfarin	0.90/0.93
Reinecke	2023	Germany	97	76.5(68–81)/77 (70.80)	35.4/24.5	5(3.5–5.0)/4.5(4–6)	NA	NA	NA	NA	NA	Apixaban 2.5 mg BID	Warfarin	1.27/1.18
Shah	2014	Canada	1,626	75.3 ± 8.1/75.1 ± 8.5	39/39	NA	NA	6/5	59/66	77/75	44/39	Warfarin	None	NA
Shen	2015	US	12,284	61.2 ± 12.4/62.1 ± 13.6	50.3/51.3	NA	NA	NA	67.3/68.3	97.2/98.6	69.1/70.8	Warfarin	None	NA
Siontis	2018	US	25,523	68.87(11.49)/68.15(11.93)	45.6/45.7	5.27 (1.77)/5.24 (1.79)	NA	NA	79.5/77.5	99.6/99.6	75.4/74.9	Apixaban 5 mg BID (44%) and 2.5 mg BID (56%)	Warfarin	NA
Sy	2022	US	11,920	74 ± 9/74 ± 9	4/4	6(5–7)/6(5–7)	NA	NA	87/88	99/99	78/79	Warfarin	None	NA
Tan	2019	US	5,765	74.4/74.7	57.0/56.8	NA	NA	13.0/13.3	68.2/69.0	98.4/98.6	70.7/71.6	Warfarin	None	NA
Vriese	2021	Belgium	90	79.9(74.4–83.9)/80.3(71.5–84.3)	23.9/43.2	4.7 (1.4)/4.8 (1.5)	NA	32.6/36.4	37/20.5	NA	43.5/45.5	Rivaroxaban 10 mg QD	Warfarin	1.88
Wakasugi	2014	Japan	60	67.8 (9.4)/68.4 (8.5)	43/28	NA	NA	NA	NA	46/50	18/28	Warfarin	None	NA
Wetmore	2022	US	17,156	NA	38.6/42.7/37.5	4.3(1.7)/4.7(1.7)/4.5 (1.7)	NA	NA	63.1/66.1/65.2	96.3/97.2/95.3	76.4/79.2/76.6	Apixaban 2.5 mg BID and 5 mg BID	Warfarin	NA
Winkelmayer	2011	US	1,185	68.9 ± 11.7/68.9 ± 12.5	58.7/56.8	NA	NA	NA	77.6/76.2	82.7/82.1	60.3/59.7	Warfarin	None	1.76
Yodogawa	2016	Japan	84	69.5 ± 10.7/70.4 ± 10.2	20/35	NA	1.7 ± 1.1/1.5 ± 1.0	10/2	20/13	57/48	37/43	Warfarin	None	3.92
Yoon	2017	Korea	9,974	67.8 ± 11.0/66.1 ± 12.6	40.1/42.5	NA	NA	NA	NA	89.4/79.2	43.1/35.9	Warfarin	None	1.33

NA, not available; CHA_2_DS_2_–VASc, congestive heart failure, hypertension, age >75 years, diabetes mellitus, history of stroke/transient ischemic attack/systemic embolism, vascular disease, age 65–74 years, female sex.

### 3.3 Quality assessment

Information on the quality and bias risk assessment of all the studies is summarized in Supplementary Table S1. Three (De Vriese et al., 2021; Pokorney et al., 2022; Reinecke et al., 2023) of the 19 studies included in this study were RCTs, and the quality evaluation is detailed in Supplementary Table S1A. Three studies (De Vriese et al., 2021; Pokorney et al., 2022; Reinecke et al., 2023) were considered to have concerns regarding bias arising during randomization. Bias due to deviation from the intended intervention and bias due to missing outcome data were considered to pose a low risk for all RCTs performed. The study by De Vriese et al. (2021) was judged to have concerns regarding bias in the selection of reported outcomes, and it was the only study with concerns regarding the overall risk of bias.

The quality evaluation results of the 16 non-randomized studies of interventions (NRSIs) (Chan et al., 2009; Shah et al., 2014; Wakasugi et al., 2014; Chan et al., 2015; Genovesi et al., 2015; Shen et al., 2015; Kai et al., 2017; Siontis et al., 2018; Mavrakanas et al., 2020; Lin et al., 2021) are detailed in Supplementary Table S1B. Among the 16 NRSIs included in this analysis, seven exhibited a moderate risk of bias due to confounding factors (Chan et al., 2009; Wakasugi et al., 2014; Genovesi et al., 2015; Yodogawa et al., 2016; Siontis et al., 2018; Tan et al., 2019; Sy et al., 2022). In terms of participant selection bias, eight studies were deemed to have a moderate risk (Wakasugi et al., 2014; Yodogawa et al., 2016; Kai et al., 2017; Yoon et al., 2017; Tan et al., 2019; Mavrakanas et al., 2020; Lin et al., 2021; Sy et al., 2022). All studies demonstrated a low risk in relation to intervention classification bias. Regarding deviations from intended interventions, four studies (Winkelmayer et al., 2011; Genovesi et al., 2015; Yodogawa et al., 2016; Sy et al., 2022) presented a moderate risk of bias. Three studies (Kai et al., 2017; Mavrakanas et al., 2020; Lin et al., 2021) were found to have a moderate risk of bias due to missing data. With respect to outcome measurement bias, two studies (Tan et al., 2019; Lin et al., 2021) were assessed as having a moderate risk, while one study (Yodogawa et al., 2016) was considered at serious risk. Six studies (Chan et al., 2009; Wakasugi et al., 2014; Chan et al., 2015; Siontis et al., 2018; Mavrakanas et al., 2020; Lin et al., 2021) were considered at low risk concerning the selection of reported results.

### 3.4 Results of the network and direct-comparison meta-analysis

#### 3.4.1 Mortality

**FIGURE 2:**
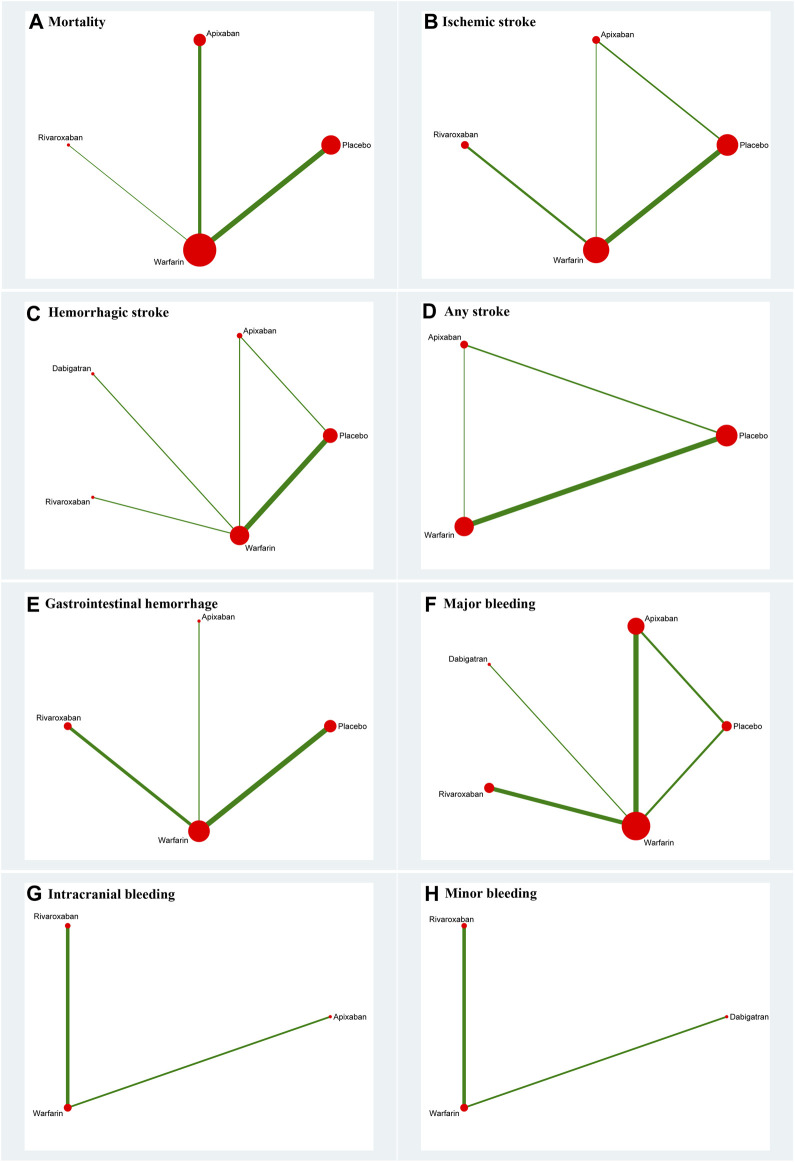
Network plot for all outcomes. **(A)** Mortality; **(B)** ischemic stroke; **(C)** hemorrhagic stroke; **(D)** any stroke; **(E)** gastrointestinal hemorrhage; **(F)** major bleeding; **(G)** intracranial bleeding; and **(H)** minor bleeding. The size of the nodes corresponds to the number of trials under study. The larger the node, the larger the number of participants in the study. The results of direct comparisons are connected by a line, the thickness of which corresponds to the sum of the sample sizes compared for each pairwise treatment. The thicker the line, the larger the sample size for comparison.

**FIGURE 3:**
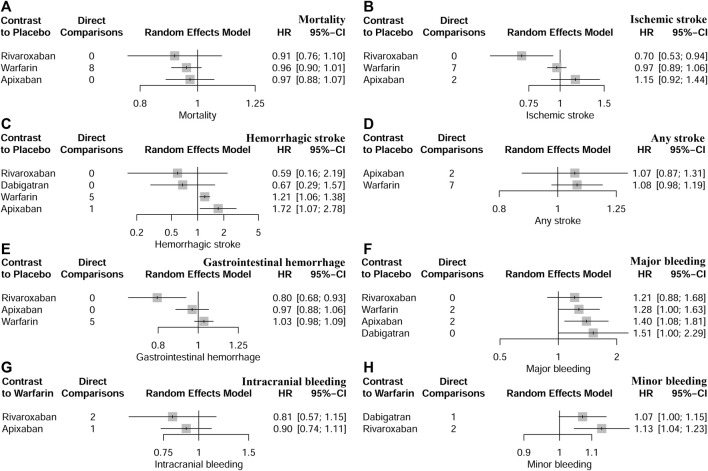
Network comparisons of anticoagulant drugs and placebo for all outcomes. **(A)** indicated mortality, **(B)** indicated ischaemic stroke, **(C)** indicated haemorrhagic stroke, **(D)** indicated any stroke, **(E)** indicated gastrointestinal haemorrhage, **(F)** indicated major bleeding, **(G)** indicated intracranial bleeding, and **(H)** indicated minor bleeding.

Thirteen studies (Chan et al., 2009; Winkelmayer et al., 2011; Wakasugi et al., 2014; Genovesi et al., 2015; Shen et al., 2015; Yodogawa et al., 2016; Kai et al., 2017; Siontis et al., 2018; Tan et al., 2019; De Vriese et al., 2021; Pokorney et al., 2022; Wetmore et al., 2022; Reinecke et al., 2023) with 62,533 participants using three anticoagulant drugs and a placebo were included in NMA to assess mortality. A network plot of mortality, including three active interventions and a placebo, is depicted in Figure 2A. Compared with placebo based on the NMA results, apixaban (HR = 0.97, 95% CI: 0.88–1.07), rivaroxaban (HR = 0.91, 95% CI: 0.76–1.10), and warfarin (HR = 0.96, 95% CI: 0.90–1.01) did not reduce mortality, as shown in Figure 3A, and comparisons of other anticoagulant drugs for mortality are summarized in Table 2. In the test quantifying overall heterogeneity, significant heterogeneity was found (I^2^ = 77.6%).

**TABLE 2:** Results of network and direct meta-analysis for mortality.

Placebo	/	1.04 (0.99 to 1.11)	/
1.09 (0.91 to 1.31)	**Rivaroxaban**	0.96 (0.80 to 1.14)	/
1.04 (0.99 to 1.11)	0.96 (0.80 to 1.14)	**Warfarin**	0.99 (0.92 to 1.06)
1.03 (0.94 to 1.13)	0.94 (0.78 to 1.14)	0.99 (0.92 to 1.06)	**Apixaban**

The lower left part represents the network comparison results, and the upper right part represents the direct comparison results. Comparison results should be interpreted from column to row; the intervention on the column is the intervention group, and the intervention on the row is the control group. Results that are in bold and underlined are statistically significant. / indicates not available.

Regarding direct comparisons of mortality, the comparisons of warfarin vs. apixaban (HR = 0.99, 95% CI: 0.92–1.06), placebo vs. warfarin (HR = 1.04, 95% CI: 0.99–1.11), and rivaroxaban vs. warfarin (HR = 0.96, 95% CI: 0.80–1.14) did not significantly reduce mortality (Table 2).

**FIGURE 4:**
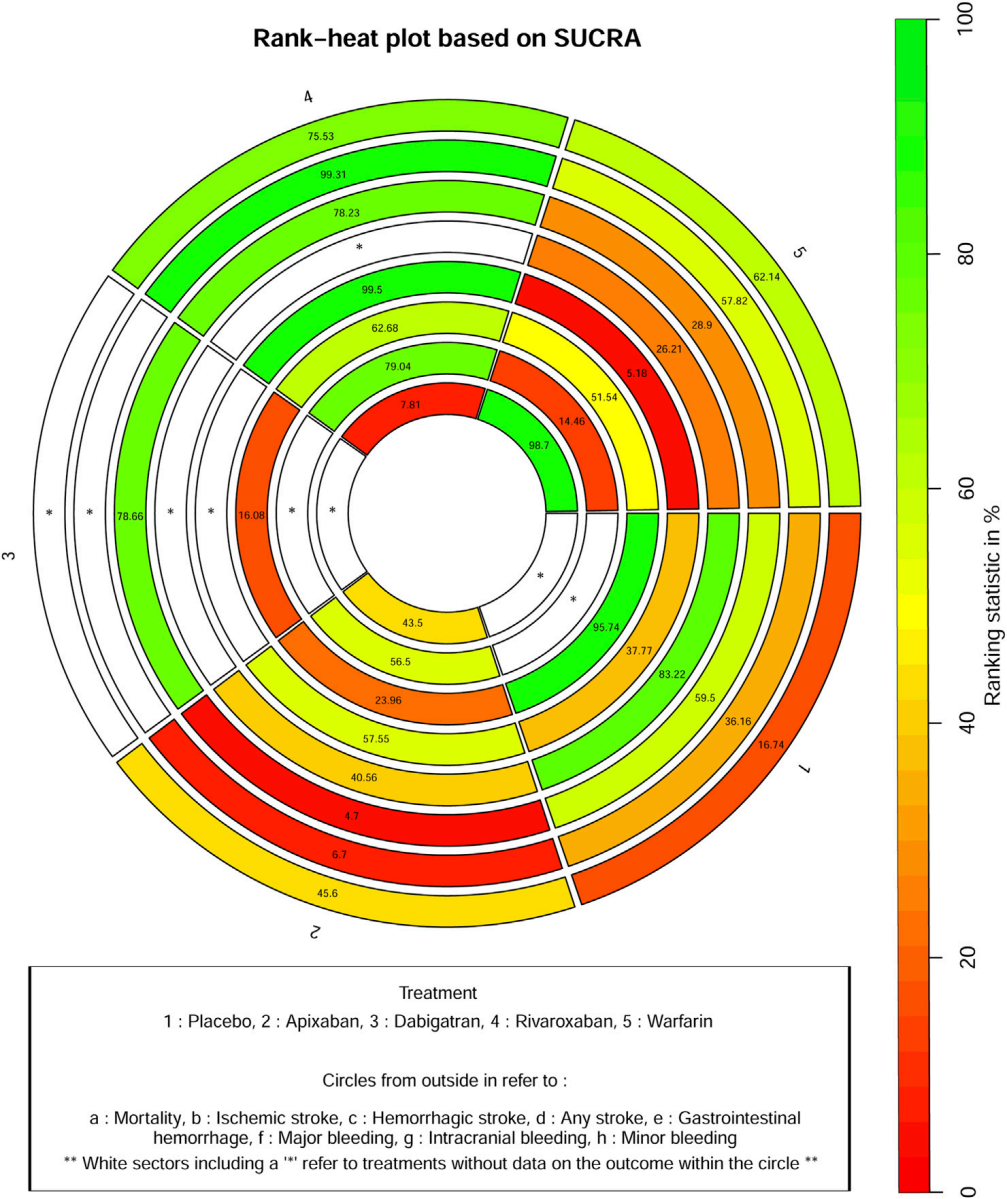
Rank−heat plot based on SUCRA for all outcomes.

Figure 4 shows that based on SUCRA, rivaroxaban (75.53%), warfarin (62.14%), and apixaban (45.6%) were the most effective interventions for managing mortality, while placebo (16.74%) was least effective.

#### 3.4.2 Ischemic stroke

Ten studies (Chan et al., 2009; Winkelmayer et al., 2011; Wakasugi et al., 2014; Shen et al., 2015; Kai et al., 2017; Tan et al., 2019; Mavrakanas et al., 2020; De Vriese et al., 2021; Lin et al., 2021; Pokorney et al., 2022) with 38,750 participants using three anticoagulant drugs and a placebo were included in NMA to assess ischemic stroke. A network plot of ischemic stroke, including three active interventions and a placebo, is depicted in Figure 2B. Compared with placebo based on the NMA results, rivaroxaban (HR = 0.70, 95% CI: 0.53–0.94) reduced the risk of ischemic stroke, while apixaban (HR = 1.15, 95% CI: 0.92–1.44) and warfarin (HR = 0.97, 95% CI: 0.89–1.06) did not reduce the risk, as shown in Figure 3B. The comparisons of other active interventions for ischemic stroke are summarized in Table 3. In the test quantifying overall heterogeneity, obvious heterogeneity was found (I^2^ = 42.0%).

**TABLE 3:** Results of network and direct meta-analysis for ischemic stroke.

Placebo	/	1.03 (0.95 to 1.13)	0.85 (0.67 to 1.06)
1.43 (1.07 to 1.90)	**Rivaroxaban**	**0.72 (0.55 to 0.95)**	/
1.03 (0.95 to 1.12)	**0.72 (0.55 to 0.95)**	**Warfarin**	1.43 (0.50 to 4.08)
0.87 (0.70 to 1.09)	**0.61 (0.42 to 0.88)**	0.84 (0.66 to 1.07)	**Apixaban**

The lower left part represents the network comparison results, and the upper right part represents the direct comparison results. Comparison results should be interpreted from column to row; the intervention on the column is the intervention group, and the intervention on the row is the control group. Results that are in bold and underlined are statistically significant. / indicates not available.

Regarding direct comparisons of ischemic stroke, the comparisons of rivaroxaban vs. warfarin (HR = 0.72, 95% CI: 0.55–0.95) showed a significantly reduced risk of ischemic stroke, and other comparisons of warfarin vs. apixaban (HR = 1.43, 95% CI: 0.50–4.08), placebo vs. warfarin (HR = 1.03, 95% CI: 0.95–1.13), and placebo vs. apixaban (HR = 0.85, 95% CI: 0.67–1.06) did not reduce the risk (Table 3).

Figure 4 shows that based on SUCRA, rivaroxaban (99.31%), warfarin (57.82%), and placebo (36.16%) were the most effective interventions for managing ischemic stroke, while apixaban (6.70%) was least effective.

#### 3.4.3 Hemorrhagic stroke

Nine studies (Chan et al., 2009; Winkelmayer et al., 2011; Chan et al., 2015; Shen et al., 2015; Kai et al., 2017; Yoon et al., 2017; Mavrakanas et al., 2020; De Vriese et al., 2021; Pokorney et al., 2022) with 32,821 participants using four anticoagulant drugs and a placebo were included in NMA to assess hemorrhagic stroke. A network plot of hemorrhagic stroke, including three active interventions and a placebo, is depicted in Figure 2C. Compared with placebo based on the NMA results, apixaban (HR = 1.72, 95% CI: 1.72–2.78) and warfarin (HR = 1.21, 95% CI: 1.06–1.38) increased the risk of hemorrhagic stroke, while rivaroxaban (HR = 0.59, 95% CI: 0.16–2.19) and dabigatran (HR = 0.67, 95% CI: 0.29–1.57) did not, as shown in Figure 3C. The comparisons of other active interventions for hemorrhagic stroke are summarized in Table 4. In the test quantifying overall heterogeneity, small heterogeneity was found (I^2^ = 9.70%).

**TABLE 4:** Results of network and direct meta-analysis for hemorrhagic stroke.

Dabigatran	/	/	0.56 (0.24 to 1.29)	/
0.67 (0.29 to 1.57)	**Placebo**	/	**0.83 (0.72 to 0.95)**	**0.59 (0.35 to 0.97)**
1.15 (0.24 to 5.43)	1.70 (0.46 to 6.35)	**Rivaroxaban**	0.49 (0.13 to 1.80)	/
0.56 (0.24 to 1.29)	**0.83 (0.72 to 0.95)**	0.49 (0.13 to 1.80)	**Warfarin**	0.66 (0.16 to 2.62)
0.39 (0.15 to 1.03)	**0.58 (0.36 to 0.93)**	0.34 (0.08 to 1.38)	0.70 (0.43 to 1.15)	**Apixaban**

The lower left part represents the network comparison results, and the upper right part represents the direct comparison results. Comparison results should be interpreted from column to row; the intervention on the column is the intervention group, and the intervention on the row is the control group. Results that are in bold and underlined are statistically significant. / indicates not available.

Regarding direct comparisons of hemorrhagic stroke, the comparisons of placebo vs. warfarin (HR = 0.83, 95% CI: 0.72–0.95) and placebo vs. apixaban (HR = 0.59, 95% CI: 0.35–0.97) revealed a significantly reduced risk of hemorrhagic stroke, but other comparisons of dabigatran vs. warfarin (HR = 0.56, 95% CI: 0.24–1.29) and dabigatran vs. warfarin (HR = 0.49, 95% CI: 0.13–1.80) did not (Table 4).

Figure 4 shows that based on SUCRA, dabigatran (78.66%), rivaroxaban (78.23%), placebo (59.50%), and warfarin (28.90%) were the most effective interventions for managing hemorrhagic stroke, while apixaban (4.70%) was least effective.

#### 3.4.4 Any stroke

Nine studies (Chan et al., 2009; Winkelmayer et al., 2011; Shah et al., 2014; Shen et al., 2015; Yodogawa et al., 2016; Siontis et al., 2018; Tan et al., 2019; Mavrakanas et al., 2020; Sy et al., 2022) with 47,346 participants using two anticoagulant drugs and a placebo were included in NMA to assess any stroke. A network plot of any stroke, including three active interventions and a placebo, is depicted in Figure 2D. Compared with placebo based on the NMA results, apixaban (HR = 1.07, 95% CI: 0.87–1.31) and warfarin (HR = 1.08, 95% CI: 0.98–1.19) did not increase the risk of any stroke, as shown in Figure 3D. The comparisons of other active interventions for any stroke are summarized in Table 5. In the test quantifying overall heterogeneity, significant heterogeneity was found (I^2^ = 68.4%).

**TABLE 5:** Results of network and direct meta-analysis for any stroke.

Placebo	0.94 (0.85 to 1.04)	0.85 (0.61 to 1.19)
0.93 (0.84 to 1.02)	**Warfarin**	1.06 (0.84 to 1.33)
0.94 (0.77 to 1.15)	1.01 (0.83 to 1.22)	**Apixaban**

The lower left part represents the network comparison results, and the upper right part represents the direct comparison results. Comparison results should be interpreted from column to row; the intervention on the column is the intervention group, and the intervention on the row is the control group. Results that are in bold and underlined are statistically significant. / indicates not available.

Regarding direct comparisons of any stroke, the comparisons of placebo vs. warfarin (HR = 0.94, 95% CI: 0.85–1.04), placebo vs. apixaban (HR = 0.85, 95% CI: 0.61–1.19), and warfarin vs. apixaban (HR = 1.06, 95% CI: 0.84–1.33) did not significantly reduce any stroke (Table 5).

Figure 4 shows that, based on SUCRA, placebo (83.22%) and apixaban (40.56%) were the most effective interventions for managing any stroke, while warfarin (26.21%) was least effective.

#### 3.4.5 Gastrointestinal hemorrhage

Eight studies (Winkelmayer et al., 2011; Shen et al., 2015; Kai et al., 2017; Yoon et al., 2017; Siontis et al., 2018; Tan et al., 2019; De Vriese et al., 2021; Lin et al., 2021) with 42,683 participants using three anticoagulant drugs and a placebo were included in NMA to assess gastrointestinal hemorrhage. A network plot of gastrointestinal hemorrhage, including three active interventions and a placebo, is depicted in Figure 2E. Compared with placebo based on the NMA results, rivaroxaban (HR = 0.80, 95% CI: 0.68–0.93) reduced the risk of gastrointestinal hemorrhage, while apixaban (HR = 0.97, 95% CI: 0.88–1.06) and warfarin (HR = 1.03, 95% CI: 0.98–1.09) did not (Figure 3E). The comparisons of other active interventions for gastrointestinal hemorrhage are summarized in Table 6. In the test quantifying overall heterogeneity, it did not find heterogeneity (I^2^ = 0%).

**TABLE 6:** Results of network and direct meta-analysis for gastrointestinal hemorrhage.

Placebo	/	0.97 (0.92 to 1.02)	/
**1.26 (1.07 to 1.48)**	**Rivaroxaban**	**0.77 (0.66 to 0.90)**	/
0.97 (0.92 to 1.02)	**0.77 (0.66 to 0.90)**	**Warfarin**	1.07 (0.99 to 1.15)
1.03 (0.94 to 1.13)	**0.82 (0.69 to 0.98)**	1.07 (0.99 to 1.15)	**Apixaban**

The lower left part represents the network comparison results, and the upper right part represents the direct comparison results. Comparison results should be interpreted from column to row; the intervention on the column is the intervention group, and the intervention on the row is the control group. Results that are in bold and underlined are statistically significant. / indicates not available.

Regarding direct comparisons of gastrointestinal hemorrhage, the comparisons of rivaroxaban vs. warfarin (HR = 0.77, 95% CI: 0.66–0.90) showed significantly reduced gastrointestinal hemorrhage, while other comparisons of placebo vs. warfarin (HR = 0.97, 95% CI: 0.92–1.02) and warfarin vs. apixaban (HR = 1.07, 95% CI: 0.99–1.15) did not (Table 6).

Figure 4 shows that based on SUCRA, rivaroxaban (99.50%), apixaban (57.55%), and placebo (37.77%) were the most effective interventions for managing gastrointestinal hemorrhage, while warfarin (5.18%) was least effective.

#### 3.4.6 Major bleeding

Eleven studies (Chan et al., 2009; Chan et al., 2015; Siontis et al., 2018; Tan et al., 2019; Mavrakanas et al., 2020; De Vriese et al., 2021; Lin et al., 2021; Pokorney et al., 2022; Reinecke et al., 2023) with 72,113 participants using four anticoagulant drugs and a placebo were included in NMA to assess major bleeding. A network plot of major bleeding, including three active interventions and a placebo, is depicted in Figure 2F. Compared with placebo based on the NMA results, apixaban (HR = 1.40, 95% CI: 1.08–1.81) increased the risk of major bleeding, while rivaroxaban (HR = 1.21, 95% CI: 0.88–1.68), warfarin (HR = 1.28, 95% CI: 1.00–1.63), and dabigatran (HR = 1.51, 95% CI: 1.00–2.29) did not, as shown in Figure 3F. The comparisons of other active interventions for major bleeding are summarized in Table 7. In the test quantifying overall heterogeneity, it found significant heterogeneity (I^2^ = 78.8%).

**TABLE 7:** Results of network and direct meta-analysis for major bleeding.

Dabigatran	/	/	1.19 (0.85 to 1.66)	/
1.51 (1.00 to 2.29)	**Placebo**	/	0.87 (0.65 to 1.18)	**0.59 (0.40 to 0.88)**
1.25 (0.84 to 1.85)	0.82 (0.60 to 1.14)	**Rivaroxaban**	0.95 (0.77 to 1.17)	/
1.19 (0.85 to 1.66)	0.78 (0.61 to 1.00)	0.95 (0.77 to 1.17)	**Warfarin**	0.95 (0.80 to 1.13)
1.08 (0.75 to 1.57)	**0.72 (0.55 to 0.93)**	0.87 (0.67 to 1.14)	0.91 (0.78 to 1.08)	**Apixaban**

The lower left part represents the network comparison results, and the upper right part represents the direct comparison results. Comparison results should be interpreted from column to row; the intervention on the column is the intervention group, and the intervention on the row is the control group. Results that are in bold and underlined are statistically significant. / indicates not available.

Regarding direct comparisons of major bleeding, the comparisons of placebo vs. apixaban (HR = 0.59, 95% CI: 0.40–0.88) showed significantly reduced major bleeding, whereas the other comparisons of warfarin vs. apixaban (HR = 0.95, 95% CI: 0.80–1.13), dabigatran vs. warfarin (HR = 1.19, 95% CI: 0.85–1.66), placebo vs. warfarin (HR = 0.87, 95% CI: 0.65–1.18), and rivaroxaban vs. warfarin (HR = 0.95, 95% CI: 0.77–1.17) did not (Table 7).

Figure 4 shows that based on SUCRA, placebo (95.74%), rivaroxaban (62.68%), and warfarin (51.54%) were the most effective interventions for managing major bleeding, while apixaban (23.96%) and dabigatran (16.08%) were least effective.

#### 3.4.7 Intracranial bleeding

Two studies (Siontis et al., 2018; Lin et al., 2021) with 16,035 participants and three anticoagulant drugs were included in NMA to assess intracranial bleeding. A network plot of intracranial bleeding, including three active interventions and a placebo, is depicted in Figure 2G. Compared with warfarin based on the NMA results, rivaroxaban (HR = 0.81, 95% CI: 0.57–1.15) and apixaban (HR = 0.90, 95% CI: 0.74–1.11) did not increase the risk of intracranial bleeding, as shown in Figure 3G. The comparisons of other active interventions for intracranial bleeding are summarized in Table 8. In the test quantifying overall heterogeneity, it did not find heterogeneity (I^2^ = 0%).

**TABLE 8:** Results of network and direct meta-analysis for intracranial bleeding.

Rivaroxaban	0.81 (0.57 to 1.15)	/
0.81 (0.57 to 1.15)	**Warfarin**	1.11 (0.90 to 1.36)
0.90 (0.59 to 1.35)	1.11 (0.90 to 1.36)	**Apixaban**

The lower left part represents the network comparison results, and the upper right part represents the direct comparison results. Comparison results should be interpreted from column to row; the intervention on the column is the intervention group, and the intervention on the row is the control group. Results that are in bold and underlined are statistically significant. / indicates not available.

Regarding direct comparisons of intracranial bleeding, the comparisons of rivaroxaban vs. warfarin (HR = 0.81, 95% CI: 0.57–1.15) and warfarin vs. apixaban (HR = 1.11, 95% CI: 0.90–1.36) did not significantly reduce intracranial bleeding (Table 8).

Figure 4 shows that based on SUCRA, rivaroxaban (79.04%) and apixaban (56.50%) were the most effective interventions for managing intracranial bleeding, while warfarin (14.46%) was least effective.

#### 3.4.8 Minor bleeding

Two studies (Chan et al., 2015; De Vriese et al., 2021) with 23,152 participants and three anticoagulant drugs were included in NMA to assess minor bleeding. A network plot of minor bleeding, including three active interventions and a placebo, is depicted in Figure 2H. Compared with warfarin based on the NMA results, rivaroxaban (HR = 1.13, 95% CI: 1.04–1.23) increased the risk of minor bleeding, while dabigatran (HR = 1.07, 95% CI: 1.00–1.15) did not, as shown in Figure 3H. The comparisons of other active interventions for minor bleeding are summarized in Table 9. In the test quantifying overall heterogeneity, it did not find heterogeneity (I^2^ = 0%).

**TABLE 9:** Results of network and direct meta-analysis for minor bleeding.

Rivaroxaban	1.13 (1.04 to 1.23)	/
**1.13 (1.04 to 1.23)**	**Warfarin**	0.93 (0.87 to 1.00)
1.06 (0.95 to 1.18)	0.93 (0.87 to 1.00)	**Dabigatran**

The lower left part represents the network comparison results, and the upper right part represents the direct comparison results. Comparison results should be interpreted from column to row; the intervention on the column is the intervention group, and the intervention on the row is the control group. Results that are in bold and underlined are statistically significant. / indicates not available.

Regarding direct comparisons of minor bleeding, the comparisons of rivaroxaban vs. warfarin (HR = 1.13, 95% CI: 1.04–1.23) showed significantly reduced minor bleeding, while those of warfarin vs. dabigatran (HR = 0.93, 95% CI: 0.87–1.00) did not (Table 9).

Figure 4 shows that based on SUCRA, warfarin (98.70%) and apixaban (43.50%) were the most effective interventions for managing minor bleeding, while rivaroxaban (7.81%) was least effective.

### 3.5 Inconsistency test

Based on separate indirect and direct evidence using the back-calculation method, inconsistencies were not found in any of the outcomes (Supplementary Table S2-9).

### 3.6 Sensitivity analysis

For sensitivity analysis, studies of Chan et al. (2015) and Yodogawa et al. (2016) with a mean heart failure rate of less than 20% were excluded in outcomes of mortality, hemorrhagic stroke, any stroke, and major bleeding. Table 10 shows that the sensitivity analysis results of reticular *versus* SUCRA were stable.

**TABLE 10:** Results of sensitivity analyses.

Anticoagulant drugs	Mortality				Hemorrhagic stroke			
	Main results		Results of sensitivity analysis		Main results		Results of sensitivity analysis	
	HR, 95% CI	SUCRA	HR, 95% CI	SUCRA	HR, 95% CI	SUCRA	HR, 95% CI	SUCRA
**Placebo**	1	16.74	1	18.62	1	59.5	1	73.16
**Apixaban**	0.97 (0.88, 1.07)	45.6	0.97 (0.89, 1.07)	44.22	1.72 (1.07, 2.78)	4.7	1.72 (1.07, 2.78)	5.06
**Dabigatran**	NA	NA	NA	NA	0.67 (0.29, 1.57)	78.66	NA	NA
**Rivaroxaban**	0.91 (0.76, 1.10)	75.53	0.92 (0.76, 1.10)	75.17	0.59 (0.16, 2.19)	78.23	0.59 (0.16, 2.19)	86.3
**Warfarin**	0.96 (0.90, 1.01)	62.14	0.96 (0.90, 1.02)	61.99	1.21 (1.07, 2.78)	28.9	1.21 (1.06, 1.38)	35.48

Anticoagulant drugs	Any stroke				Major bleeding			
	Main results		Results of sensitivity analysis		Main results		Results of sensitivity analysis	
	HR, 95% CI	SUCRA	HR, 95% CI	SUCRA	HR, 95% CI	SUCRA	HR, 95% CI	SUCRA
**Placebo**	1	83.22	1	83.36	1	95.74	1	88.3
**Apixaban**	1.07 (0.87, 1.31)	40.56	1.07 (0.87, 1.31)	39.38	1.40 (1.08, 1.81)	23.96	1.39 (1.08, 1.80)	5.81
**Dabigatran**	NA	NA	NA	NA	1.51 (1.00, 2.29)	16.08	NA	NA
**Rivaroxaban**	NA	NA	NA	NA	1.21 (0.88, 1.68)	62.68	1.08 (0.76, 1.55)	71.96
**Warfarin**	1.08 (0.98, 1.19)	26.21	1.08 (0.98, 1.19)	27.27	1.28 (1.00, 1.63)	51.54	1.28 (1.00, 1.62)	33.93

The results of HRs are network comparisons of anticoagulant drugs and placebo. CIs, confidence intervals; HRs, hazard ratios; NA, not available; SUCRA, surface under the cumulative ranking curve. Significant results are in bold.

### 3.7 Publication bias

Publication bias was not found in any of the network funnel plots, as shown in Supplementary Table S1-8.

## 4 Discussion

AF was a prevalent arrhythmia among ESRD patients undergoing dialysis (Chan and Siu, 2015). Both non-valvular AF and ESRD were independent risk factors for stroke and mortality (Bonde et al., 2015). However, the utilization of DOACs in ESRD patients with AF who undergo dialysis remained a controversial subject (Yao et al., 2016). Previous analyses had demonstrated that patients with AF undergoing dialysis were at a significantly elevated risk of stroke and bleeding when compared to those who did not receive anticoagulation therapy (Almutairi et al., 2017). The utilization of DOACs has been consistently increasing in recent years (Caldeira et al., 2015). However, currently, there is a lack of direct head-to-head comparisons between different DOACs. The lack of comprehensive evidence had posed a challenge for clinicians in regard to recommending one oral anticoagulant over another. To gain further insights into this issue, this study conducted a network meta-analysis to evaluate the efficacy of individual oral anticoagulants. This systematic review and meta-analysis of 19 studies revealed no significant differences in mortality, any stroke, or intracranial hemorrhage among rivaroxaban, warfarin, apixaban, and placebo. However, the network meta-analysis demonstrated that rivaroxaban treatment results in a lower incidence of ischemic stroke and gastrointestinal bleeding than did other treatments but causes an increased risk of minor bleeding events. Apixaban and warfarin were associated with an increased risk of hemorrhagic stroke, while apixaban additionally posed a heightened risk of major bleeding. Conventional meta-analysis findings indicate that placebo administration can effectively reduce the incidence of hemorrhagic stroke. Based on SUCRA, rivaroxaban demonstrated superior efficacy in mitigating mortality, ischemic stroke, gastrointestinal bleeding, and intracranial hemorrhage. The most significant reduction in hemorrhagic stroke was observed with dabigatran. Placebo therapy demonstrated the highest efficacy in reducing the incidence of any type of stroke and major bleeding, while warfarin proved to be most effective in reducing minor bleeding.

Patients with ESRD and AF undergoing dialysis had a high mortality rate, but a significant proportion of deaths were not attributable to cardiovascular events. Both the network and traditional meta-analysis indicated that the use of DOACs did not reduce mortality, which was consistent with the literature. In a study (Pan et al., 2017) examining dialysis recipients with AF, DOAC therapy did not lead to a reduction in the risk of all-cause mortality. Furthermore, a meta-analysis (Cohen et al., 2015) encompassing 12 cohort studies involving 17,380 participants revealed that warfarin had no significant impact on mortality among AF patients undergoing hemodialysis. Notably, when considering the comprehensive ranking using SUCRA analysis, rivaroxaban exhibited the greatest reduction in mortality, while apixaban demonstrated the least effect. There was insufficient evidence to support the reduction in mortality with rivaroxaban compared to other DOACs in patients with ESRD and AF undergoing dialysis (Geurts et al., 2022). However, the conclusion appeared plausible based on pharmacokinetic and pharmacodynamic studies demonstrating that a 10-mg dose of rivaroxaban in ESRD patients achieved similar drug levels as a 20-mg dose in healthy individuals, and it was not eliminated through dialysis in ESRD patients (Wetmore et al., 2022). The precise relationship between apixaban and mortality remained uncertain. However, evidence suggested a dose-dependent association, with lower doses of apixaban potentially linked to higher mortality rates. While the standard 5-mg dosage of apixaban was associated with reduced mortality, the administration of a 2.5-mg dose appeared to be correlated with an increase in mortality.

The incidence of AF was significantly elevated in ESRD patients receiving dialysis. A study conducted by Vazquez et al. (2016) revealed that the occurrence of new-onset AF in CKD patients undergoing dialysis was significantly associated with a nine-fold increased risk of stroke. Furthermore, a meta-analysis comprising 21 prospective studies demonstrated an inverse relationship between eGFR <60 mL/min/1.73 m^2^ and a significantly elevated risk of stroke (Harenberg et al., 2012). An observational study of a randomly selected sample comprising 17,518 patients with ESRD undergoing hemodialysis demonstrated that AF was associated with a 1.8-fold increased risk of stroke (Skjøth et al., 2014). Another investigation revealed the presence of proteinuria thromboembolism by 1.5-fold after adjusting for established stroke risk factors and other potential confounders. The network meta-analysis conducted in this study revealed that rivaroxaban significantly reduced the incidence of ischemic stroke, while apixaban and warfarin were found to be associated with an increased incidence of hemorrhagic stroke. The efficacy of apixaban in reducing thromboembolism had been demonstrated, with a dosage of 5 mg administered twice daily showing superior results compared to warfarin. In accordance with the KDIGO 2012 guidelines (Gordon et al., 2023), utilizing a lower dosage of 2.5 mg apixaban for stroke prevention in patients with ESKD and AF was tentatively considered. This observed discrepancy arose from previous studies that combined ischemic stroke and hemorrhagic stroke as a single outcome. Rates of stroke did not show any significant association with the use of DOACs. Given that vascular factors were the primary cause of stroke in patients with ESRD, the potential benefits of anticoagulant therapy in reducing stroke risk were limited. Conventional meta-analyses had suggested that the non-use of anticoagulant therapy could potentially decrease the incidence of hemorrhagic strokes. Compared to the non-use of anticoagulant therapy, the use of warfarin-based DOAC therapy had been suggested to confer significant benefits in the prevention of thromboembolic events and all-cause mortality, without an increased risk of bleeding events.

According to the SUCRA comprehensive ranking, rivaroxaban demonstrated the greatest reduction in ischemic stroke, while apixaban showed the least. Dabigatran was found to be most effective in reducing hemorrhagic stroke incidence, whereas apixaban was the least effective. Placebo exhibited the highest efficacy in reducing any type of stroke, with warfarin being the most effective in managing minor bleeding. According to the AHA/ACC/HRS guidelines (January et al., 2019), for patients with non-valvular AF and CHA_2_DS_2_-VASc scores of 2 or higher and end-stage CKD (creatinine clearance <15 mL/min) or who were on dialysis, warfarin (INR 2.0–3.0) or apixaban was recommended. However, Chan and Siu (2015) found that, in patients with AF, warfarin use was associated with a significantly increased risk of new stroke compared to non-use of warfarin. The more detailed conclusion was that there was no observed association between warfarin and ischemic stroke in patients with ESRD, but an association existed between warfarin and an increased risk of hemorrhagic stroke in patients with AF. In summary, the findings of this study supported the notion that warfarin did not confer any benefit in reducing the incidence of stroke among ESRD patients receiving dialysis, aligning with the overall conclusion drawn from this investigation.

In our network meta-analysis, rivaroxaban demonstrated a reduction in the risk of gastrointestinal bleeding. However, it was associated with an increased risk of minor bleeding. Conversely, apixaban was found to increase the risk of major bleeding. Neither network nor traditional meta-analysis indicated that DOACs were ineffective in reducing intracranial hemorrhage. Consistent with the present study (Buckley et al., 2022), the use of DOACs did not yield beneficial outcomes in terms of reducing bleeding rates among ESRD patients with AF undergoing dialysis. Among patients receiving dialysis and diagnosed with AF, treatment with DOACs was associated with a 28% increased risk of bleeding events. In a meta-analysis comprising 12 cohort studies involving 17,380 participants, the use of warfarin in AF patients undergoing hemodialysis was associated with a 21% higher risk of bleeding (Briere et al., 2019). According to the SUCRA comprehensive ranking, the administration of rivaroxaban significantly decreased the incidence of gastrointestinal and intracranial bleeding to the greatest extent, while warfarin exhibited the least favorable outcomes. Conversely, minor bleeding showed the opposite pattern. The rate of major bleeding was most effectively reduced by placebo and least effectively reduced by dabigatran. A study utilizing a US insurance claims database demonstrated that in AF patients with ESRD, the risk of ischemic stroke/systemic embolism was associated with a lower risk of major bleeding when the patients were treated with rivaroxaban compared to warfarin (Lip et al., 2016). Siontis et al. (2018) investigated 2,351 AF patients with ESRD receiving apixaban and 23,172 receiving warfarin and concluded that apixaban significantly decreased the risk of major bleeding. Notably, the annual bleeding rate, particularly intracranial bleeding, was found to be high for both apixaban and warfarin in this study, with more than two-thirds of patients discontinuing DOACs within 1 year.

Furthermore, it was worth mentioning that DOACs exhibit reduced efficacy in mitigating bleeding events among ESRD patients with AF undergoing dialysis, which may be attributed to the association between CKD and an increased risk of bleeding. Platelet dysfunction in individuals with ESRD, impaired renal clearance, and concurrent heparin use during hemodialysis collectively heightened the susceptibility to bleeding among dialysis recipients utilizing DOACs. Considering that hemodialysis is usually conducted three times per week, administering DOACs once or twice daily may lead to drug accumulation over the extended intervals between treatment sessions (Kumar et al., 2019). Later, as drug accumulation occurs, the efficacy of DOACs diminishes, exacerbating bleeding in parallel with deteriorating renal function. CKD patients undergoing oral anticoagulant therapy were susceptible to glomerular hemorrhage and renal tubular obstruction due to excessive anticoagulation, as well as anticoagulant-related nephropathy that further compromised renal function. Moreover, when considering the utilization of DOACs in ESRD patients, it was crucial to account for the degree of decline in renal function since studies had demonstrated heterogeneity across CKD stages concerning all-cause mortality, thromboembolic events, and bleeding incidents.

A nationwide cohort study conducted in 2016 identified DOACs as safe and effective alternatives to warfarin therapy, demonstrating their potential for clinical use (Larsen et al., 2016). Regarding the prevention of ischemic stroke alone, no significant disparities were observed between DOACs and warfarin. However, when considering the combined endpoint of ischemic stroke and systemic embolism, rivaroxaban posed a risk lower than that posed by warfarin. In contrast, the effects of dabigatran and apixaban were not statistically significant. Apixaban and dabigatran were associated with a decreased risk of mortality compared to rivaroxaban or warfarin. Meanwhile, a 2021 meta-analysis concluded that apixaban and rivaroxaban may serve as potential alternatives to warfarin anticoagulants because they did not increase the risk of major bleeding or stroke (Abdullah et al., 2021). In contrast, See et al. (2021) concluded that DOACs were not more favorable than warfarin in terms of efficacy and safety in patients with AF on dialysis. The use of DOACs was not associated with a reduced risk of ischemic stroke/systemic embolism in patients with ESRD combined with AF. A network meta-analysis from 2021 also suggested that DOACs were superior to warfarin in preventing thromboembolic events and reducing bleeding risk in AF patients with glomerular filtration rates of 15–60 mL/min (Su et al., 2021). However, further high-quality evidence was still required to establish tailored treatment regimens for DOACs in diverse populations.

### Clinical implication

This NMA examined the previous three RCTs and sixteen observational cohorts of anticoagulant drugs and compared the effects of four anticoagulant drugs used for patients with atrial fibrillation on dialysis. Our study demonstrated that the effectiveness and safety of these four anticoagulant drugs had different disadvantages and advantages. Among them, rivaroxaban showed relatively better efficacy and safety than the remaining anticoagulant drugs. This contrasts with the latest AHA/ACC/HRS guidelines (January et al., 2019), which recommend warfarin and apixaban for patients with non-valvular AF on dialysis. Therefore, the decision evaluation based on NMA in this study provided new evidence for guidelines and clinicians, offering new insights into the use of anticoagulant therapy in therapeutic regimens for AF patients on dialysis.

#### Advantages and limitations

This meta-analysis had several advantages. First, NMA and conventional meta-analysis were employed in this study; they confirmed each other and enhanced the strength of the evidence. Previous studies, including RCTs and meta-analyses, provided conflicting conclusions, and our study confirmed their points of contradiction. Second, studies with a heart failure rate of less than 20% were excluded; sensitivity analysis showed that the network results were stable and confirmed the reliability and credibility of our evidence. Third, in the absence of direct head-to-head studies between various DOACs, warfarin, and placebo, this study compared and ranked the effectiveness of anticoagulant drugs used for patients with AF on dialysis using NMA, which provided a theoretical basis for clinical staff to select the anticoagulant drugs.

This study had several limitations. Regarding the data analysis, the presence of statistical heterogeneity in the outcome analyses and the inherent clinical and methodological heterogeneity may have exerted an influence on our findings. Our study employed an intention-to-treat design and did not account for changes or discontinuation of DOACs, leading to variations in patient categorization. Furthermore, both adjusted and unadjusted outcomes were amalgamated in observational studies, which could have impacted our results. In most studies, the incidence rate of events was low, and the 95% CI of the effect measure was wide. The network structure was highly sparse, resulting in limited power for consistency testing and minimal opportunity for cycle testing. Consequently, it was not feasible to estimate differences between models in a network meta-analysis. Regarding the study design, various DOACs exhibited varying degrees of renal excretion. Therefore, decisions regarding DOAC selection or dosage may have been influenced by individual patient characteristics such as renal function and weight, which could have introduced potential bias. The inclusion of studies did not explicitly mention the patients’ prior use of oral anticoagulants, which also introduced lead time bias into the study. Furthermore, it was not feasible to conduct stratified subgroup analyses based on varying follow-up durations and different selection criteria.

## 5 Conclusion

In conclusion, rivaroxaban demonstrated efficacy in reducing mortality and the incidence of ischemic stroke, gastrointestinal bleeding, and intracranial hemorrhage. Dabigatran was recommended for the prevention of hemorrhagic strokes. However, caution should be exercised due to the risk of major bleeding. Warfarin could effectively reduce minor bleeding but did not offer significant protection against gastrointestinal or intracranial bleeding. Apixaban was not recommended for mortality reduction or for preventing ischemic or hemorrhagic strokes. Further RCTs are warranted to establish specific clinical protocols.

## Data Availability

The original contributions presented in the study are included in the article/Supplementary Material; further inquiries can be directed to the corresponding author.
