# Supplementary material for: Anticoagulant drugs for patients with atrial fibrillation on dialysis: a systematic analysis and network meta-analysis
Source: Front Pharmacol. 2023 Dec 15;14:1320939. doi: 10.3389/fphar.2023.1320939 (PMC10755581; doi:10.3389/fphar.2023.1320939)

**Supplementary Material**

**Supplementary Method 1** Search strategy

**Supplementary Table 1** The quality and bias risk assessment of all studies

**Supplementary Table 2** Inconsistency test of mortality

**Supplementary Table 3** Inconsistency test of ischemic stroke

**Supplementary Table 4** Inconsistency test of hemorrhagic stroke

**Supplementary Table 5** Inconsistency test of any stroke

**Supplementary Table 6** Inconsistency test of gastrointestinal hemorrhage

**Supplementary Table 7** Inconsistency test of major bleeding

**Supplementary Table 8** Inconsistency test of intracranial bleeding

**Supplementary Table 9** Inconsistency test of minor bleeding

**Supplementary Figure 1** Funnel plot of mortality

**Supplementary Figure 2** Funnel plot of ischemic stroke

**Supplementary Figure 3** Funnel plot of hemorrhagic stroke

**Supplementary Figure 4** Funnel plot of any stroke

**Supplementary Figure 5** Funnel plot of gastrointestinal hemorrhage

**Supplementary Figure 6** Funnel plot of major bleeding

**Supplementary Figure 7** Funnel plot of intracranial bleeding

**Supplementary Figure 8** Funnel plot of minor bleeding

**Supplementary Method 1** Search strategy

1. **PubMed, February 2, 2023**

("renal dialysis"[MeSH Terms] OR "haemodialysis"[All Fields] OR "renal dialysis"[All Fields] OR "hemodialysis"[All Fields] OR "dialysis"[All Fields] OR "dialysis"[MeSH Terms] OR "renal insufficiency, chronic"[MeSH Terms] OR "chronic renal insufficiency"[All Fields] OR "chronic kidney disease"[All Fields] OR "kidney diseases"[All Fields] OR "kidney disease"[All Fields] OR "renal insufficiency"[All Fields] OR "chronic kidney failure"[All Fields] OR "end stage renal disease"[All Fields] OR "chronic kidney failure"[All Fields] OR "end stage kidney disease"[All Fields]) AND ("Atrial Fibrillation"[MeSH Terms] OR "Auricular Fibrillation"[All Fields] OR "Fibrillation, Paroxysmal Atrial"[All Fields] OR "Paroxysmal Atrial Fibrillation"[All Fields]) AND (((((((((((("dabigatran"[MeSH Terms] OR "dabigatran"[All Fields]) AND ("edoxaban"[Supplementary Concept] OR "edoxaban"[All Fields])) OR (("dabigatran"[MeSH Terms] OR "dabigatran"[All Fields]) AND ("rivaroxaban"[MeSH Terms] OR "rivaroxaban"[All Fields]))) OR (("dabigatran"[MeSH Terms] OR "dabigatran"[All Fields]) AND ("apixaban"[Supplementary Concept] OR "apixaban"[All Fields]))) OR (("dabigatran"[MeSH Terms] OR "dabigatran"[All Fields]) AND ("warfarin"[MeSH Terms] OR "warfarin"[All Fields]))) OR (("edoxaban"[Supplementary Concept] OR "edoxaban"[All Fields]) AND ("rivaroxaban"[MeSH Terms] OR "rivaroxaban"[All Fields]))) OR (("edoxaban"[Supplementary Concept] OR "edoxaban"[All Fields]) AND ("apixaban"[Supplementary Concept] OR "apixaban"[All Fields]))) OR (("edoxaban"[Supplementary Concept] OR "edoxaban"[All Fields]) AND ("warfarin"[MeSH Terms] OR "warfarin"[All Fields]))) OR (("rivaroxaban"[MeSH Terms] OR "rivaroxaban"[All Fields]) AND ("apixaban"[Supplementary Concept] OR "apixaban"[All Fields]))) OR (("rivaroxaban"[MeSH Terms] OR "rivaroxaban"[All Fields]) AND ("warfarin"[MeSH Terms] OR "warfarin"[All Fields]))) OR (("apixaban"[Supplementary Concept] OR "apixaban"[All Fields]) AND ("warfarin"[MeSH Terms] OR "warfarin"[All Fields]))) OR ("dabigatran"[MeSH Terms] OR "dabigatran"[All Fields]) AND ("oral"[All Fields] AND ("anticoagulants"[Pharmacological Action] OR "anticoagulants"[MeSH Terms] OR "anticoagulants"[All Fields] OR "anticoagulant"[All Fields])) OR ("edoxaban"[Supplementary Concept] OR "edoxaban"[All Fields]) AND ("oral"[All Fields] AND ("anticoagulants"[Pharmacological Action] OR "anticoagulants"[MeSH Terms] OR "anticoagulants"[All Fields] OR "anticoagulant"[All Fields])) OR ("rivaroxaban"[MeSH Terms] OR "rivaroxaban"[All Fields]) AND ("oral"[All Fields] AND ("anticoagulants"[Pharmacological Action] OR "anticoagulants"[MeSH Terms] OR "anticoagulants"[All Fields] OR "anticoagulant"[All Fields])) OR ("apixaban"[Supplementary Concept] OR "apixaban"[All Fields]) AND ("oral"[All Fields] AND ("anticoagulants"[Pharmacological Action] OR "anticoagulants"[MeSH Terms] OR "anticoagulants"[All Fields] OR "anticoagulant"[All Fields])) OR (("warfarin"[MeSH Terms] OR "warfarin"[All Fields]) AND ("oral"[All Fields] AND ("anticoagulants"[Pharmacological Action] OR "anticoagulants"[MeSH Terms] OR "anticoagulants"[All Fields] OR "anticoagulant"[All Fields])))) /(374)

1. **EMBASE, February 2, 2023**

('hemodialysis'/exp OR hemodialysis OR 'dialysis'/exp OR dialysis OR 'chronic kidney failure'/exp OR 'chronic kidney failure' OR 'end stage renal disease'/exp OR 'end stage renal disease') AND ('Atrial Fibrillation'/exp OR 'Auricular Fibrillation' OR 'Fibrillation, Paroxysmal Atrial' OR 'Paroxysmal Atrial Fibrillation') AND (dabigatran AND rivaroxaban OR (dabigatran AND edoxaban) OR (dabigatran AND apixaban) OR (dabigatran AND warfarin) OR (dabigatran AND 'anticoagulant agent') OR (edoxaban AND 'anticoagulant agent') OR (edoxaban AND rivaroxaban) OR (edoxaban AND apixaban) OR (edoxaban AND warfarin) OR (rivaroxaban AND warfarin) OR (rivaroxaban AND apixaban) OR (rivaroxaban AND 'anticoagulant agent') OR (apixaban AND 'anticoagulant agent') OR (apixaban AND warfarin) OR ('anticoagulant agent' AND warfarin)) /(1320)

1. **Cochrane Library, Issue 2 of 12, February 2023**

((haemodialysis OR renal dialysis OR (kidney diseases) OR (kidney disease) OR (renal insufficiency) OR (kidney failure) OR (end stage renal disease) OR (end stage kidney disease))) AND ((Atrial Fibrillation) OR (Auricular Fibrillation) OR (Fibrillation, Paroxysmal Atrial) OR (Paroxysmal Atrial Fibrillation)) AND ((dabigatran AND rivaroxaban) OR (dabigatran AND edoxaban) OR (dabigatran AND apixaban) OR (dabigatran AND warfarin) OR (dabigatran AND 'anticoagulant agent') OR (edoxaban AND 'anticoagulant agent') OR (edoxaban AND rivaroxaban) OR (edoxaban AND apixaban) OR (edoxaban AND warfarin) OR (rivaroxaban AND warfarin) OR (rivaroxaban AND apixaban) OR (rivaroxaban AND 'anticoagulant agent') OR (apixaban AND 'anticoagulant agent') OR (apixaban AND warfarin) OR ('anticoagulant agent' AND warfarin)) /(119)

**Supplementary Table 1:** The quality and bias risk assessment of all studies

**Supplementary Table 1A:** Risk of bias for randomized controlled trials

| **Study** | **Year** | **Bias arising from the randomization process** | **Bias due to deviations from intended interventions** | **Bias due to missing outcome data** | **Bias in measurement of the outcome** | **Bias in selection of the reported result** | **Overall risk of bias** |
| --- | --- | --- | --- | --- | --- | --- | --- |
| Pokorney | 2022 | Some concerns | Low risk | Low risk | Low risk | Low risk | Low risk |
| Reinecke | 2023 | Low risk | Low risk | Low risk | Low risk | Low risk | Low risk |
| Vriese | 2021 | Some concerns | Low risk | Low risk | Low risk | Some concerns | Some concerns |

**Supplementary Table 1B:** Risk of bias for non-randomized controlled trials

| **Study** | **Year** | **Bias due to confounding** | **Bias in selection of participants into the study** | **Bias in classification of interventions** | **Bias due to deviations from intended interventions** | **Bias due to missing data** | **Bias in measurement of outcomes** | **Bias in selection of the reported result** |
| --- | --- | --- | --- | --- | --- | --- | --- | --- |
| Chan | 2009 | Moderate risk | Low risk | Low risk | Low risk | Low risk | Low risk | Low risk |
| Chan | 2015 | Low risk | Low risk | Low risk | Low risk | Low risk | Low risk | Low risk |
| Genovesi | 2015 | Moderate risk | Low risk | Low risk | Moderate risk | Low risk | Low risk | Moderate risk |
| Kai | 2017 | Low risk | Moderate risk | Low risk | Low risk | Moderate risk | Low risk | Moderate risk |
| Lin | 2021 | Low risk | Moderate risk | Low risk | Low risk | Moderate risk | Moderate risk | Low risk |
| Mavrakanas | 2020 | Low risk | Moderate risk | Low risk | Low risk | Moderate risk | Low risk | Low risk |
| Shah | 2014 | Low risk | Low risk | Low risk | Low risk | Low risk | Low risk | Moderate risk |
| Shen | 2015 | Low risk | Low risk | Low risk | Low risk | Low risk | Low risk | Moderate risk |
| Siontis | 2018 | Moderate risk | Low risk | Low risk | Low risk | Low risk | Low risk | Low risk |
| Sy | 2022 | Moderate risk | Moderate risk | Low risk | Moderate risk | Low risk | Low risk | Moderate risk |
| Tan | 2019 | Moderate risk | Moderate risk | Low risk | Low risk | Low risk | Moderate risk | Moderate risk |
| Wakasugi | 2014 | Moderate risk | Moderate risk | Low risk | Low risk | Low risk | Low risk | Low risk |
| Wetmore | 2022 | Low risk | Low risk | Low risk | Low risk | Low risk | Low risk | Moderate risk |
| Winkelmayer | 2011 | Low risk | Low risk | Low risk | Moderate risk | Low risk | Low risk | Moderate risk |
| Yodogawa | 2016 | Moderate risk | Moderate risk | Low risk | Moderate risk | Low risk | Serious risk | Moderate risk |
| Yoon | 2017 | Low risk | Moderate risk | Low risk | Low risk | Low risk | Low risk | Moderate risk |

**Supplementary Table 2** Inconsistency test of mortality

| **Comparison** | **k** | **prop** | **nma** | **direct** | **indirect** | **ROR** | **z** | **P-value** |
| --- | --- | --- | --- | --- | --- | --- | --- | --- |
| Placebo:Apixaban | 0 | 0 | 1.03 | . | 1.03 | . | . | . |
| Rivaroxaban:Apixaban | 0 | 0 | 0.94 | . | 0.94 | . | . | . |
| Warfarin:Apixaban | 5 | 1.00 | 0.99 | 0.99 | . | . | . | . |
| Placebo:Rivaroxaban | 0 | 0 | 1.09 | . | 1.09 | . | . | . |
| Placebo:Warfarin | 8 | 1.00 | 1.04 | 1.04 | . | . | . | . |
| Rivaroxaban:Warfarin | 1 | 1.00 | 0.96 | 0.96 | . | . | . | . |

**Note:** K: Number of studies providing direct evidence, prop: Direct evidence proportion, nma: Estimated treatment effect (HR) in network meta-analysis, direct: Estimated treatment effect (HR) derived from direct evidence, indirect: Estimated treatment effect (HR) derived from indirect evidence, RoR: Ratio of Ratios (direct versus indirect), z: z-value of test for disagreement (direct versus indirect), P-value: P-value of test for disagreement (direct versus indirect).

**Supplementary Table 3** Inconsistency test of ischemic stroke

| **Comparison** | **k** | **prop** | **nma** | **direct** | **indirect** | **ROR** | **z** | **P-value** |
| --- | --- | --- | --- | --- | --- | --- | --- | --- |
| Placebo:Apixaban | 2 | 0.96 | 0.87 | 0.85 | 1.48 | 0.57 | -1.01 | 0.3113 |
| Rivaroxaban:Apixaban | 0 | 0 | 0.61 | . | 0.61 | . | . | . |
| Warfarin:Apixaban | 1 | 0.05 | 0.84 | 1.43 | 0.82 | 1.74 | 1.01 | 0.3113 |
| Placebo:Rivaroxaban | 0 | 0 | 1.43 | . | 1.43 | . | . | . |
| Placebo:Warfarin | 7 | 0.99 | 1.03 | 1.03 | 0.59 | 1.74 | 1.01 | 0.3113 |
| Rivaroxaban:Warfarin | 3 | 1.00 | 0.72 | 0.72 | . | . | . | . |

**Note:** K: Number of studies providing direct evidence, prop: Direct evidence proportion, nma: Estimated treatment effect (HR) in network meta-analysis, direct: Estimated treatment effect (HR) derived from direct evidence, indirect: Estimated treatment effect (HR) derived from indirect evidence, RoR: Ratio of Ratios (direct versus indirect), z: z-value of test for disagreement (direct versus indirect), P-value: P-value of test for disagreement (direct versus indirect).

**Supplementary Table 4** Inconsistency test of hemorrhagic stroke

| **Comparison** | **k** | **prop** | **nma** | **direct** | **indirect** | **ROR** | **z** | **P-value** |
| --- | --- | --- | --- | --- | --- | --- | --- | --- |
| Dabigatran:Apixaban | 0 | 0 | 0.39 | . | 0.39 | . | . | . |
| Placebo:Apixaban | 1 | 0.88 | 0.58 | 0.59 | 0.54 | 1.08 | 0.10 | 0.9193 |
| Rivaroxaban:Apixaban | 0 | 0 | 0.34 | . | 0.34 | . | . | . |
| Warfarin:Apixaban | 1 | 0.13 | 0.70 | 0.66 | 0.71 | 0.93 | -0.10 | 0.9193 |
| Dabigatran:Placebo | 0 | 0 | 0.67 | . | 0.67 | . | . | . |
| Dabigatran:Rivaroxaban | 0 | 0 | 1.15 | . | 1.15 | . | . | . |
| Dabigatran:farfarin | 1 | 1.00 | 0.56 | 0.56 | . | . | . | . |
| Wone:Rivaroxabban | 0 | 0 | 1.70 | . | 1.70 | . | . | . |
| Placebo:Warfarin | 5 | 0.99 | 0.83 | 0.83 | 0.89 | 0.93 | -0.10 | 0.9193 |
| Rivaroxaban:Narfarin | 1 | 1.00 | 0.49 | 0.49 | . | . | . | . |

**Note:** K: Number of studies providing direct evidence, prop: Direct evidence proportion, nma: Estimated treatment effect (HR) in network meta-analysis, direct: Estimated treatment effect (HR) derived from direct evidence, indirect: Estimated treatment effect (HR) derived from indirect evidence, RoR: Ratio of Ratios (direct versus indirect), z: z-value of test for disagreement (direct versus indirect), P-value: P-value of test for disagreement (direct versus indirect).

**Supplementary Table 5** Inconsistency test of any stroke

| **Comparison** | **k** | **prop** | **nma** | **direct** | **indirect** | **ROR** | **z** | **P-value** |
| --- | --- | --- | --- | --- | --- | --- | --- | --- |
| Placebo:Apixaban | 2 | 0.37 | 0.94 | 0.85 | 0.99 | 0.86 | -0.70 | 0.4815 |
| Warfarin:Apixaban | 1 | 0.69 | 1.01 | 1.06 | 0.91 | 1.16 | 0.70 | 0.4815 |
| Placebo:Warfarin | 7 | 0.94 | 0.93 | 0.94 | 0.81 | 1.16 | 0.70 | 0.4815 |

**Note:** K: Number of studies providing direct evidence, prop: Direct evidence proportion, nma: Estimated treatment effect (HR) in network meta-analysis, direct: Estimated treatment effect (HR) derived from direct evidence, indirect: Estimated treatment effect (HR) derived from indirect evidence, RoR: Ratio of Ratios (direct versus indirect), z: z-value of test for disagreement (direct versus indirect), P-value: P-value of test for disagreement (direct versus indirect).

**Supplementary Table 6** Inconsistency test of gastrointestinal hemorrhage

| **Comparison** | **k** | **prop** | **nma** | **direct** | **indirect** | **ROR** | **z** | **P-value** |
| --- | --- | --- | --- | --- | --- | --- | --- | --- |
| Placebo:Apixabban | 0 | 0 | 1.03 | . | 1.03 | . | . | . |
| Rivaroxaban:Apixaban | 0 | 0 | 0.82 | . | 0.82 | . | . | . |
| Warfarin:Apixaban | 1 | 1.00 | 1.07 | 1.07 | . | . | . | . |
| Placebo:Rivaroxaban | 0 | 0 | 1.26 | . | 1.26 | . | . | . |
| Placebo:Warfarin | 5 | 1.00 | 0.97 | 0.97 | . | . | . | . |
| Rivaroxaban:Warfarin | 3 | 1.00 | 0.97 | 0.77 | . | . | . | . |

**Note:** K: Number of studies providing direct evidence, prop: Direct evidence proportion, nma: Estimated treatment effect (HR) in network meta-analysis, direct: Estimated treatment effect (HR) derived from direct evidence, indirect: Estimated treatment effect (HR) derived from indirect evidence, RoR: Ratio of Ratios (direct versus indirect), z: z-value of test for disagreement (direct versus indirect), P-value: P-value of test for disagreement (direct versus indirect).

**Supplementary Table 7** Inconsistency test of major bleeding

| **Comparison** | **k** | **prop** | **nma** | **direct** | **indirect** | **ROR** | **z** | **P-value** |
| --- | --- | --- | --- | --- | --- | --- | --- | --- |
| Dabigatran:Apixaban | 0 | 0 | 1.08 | . | 1.08 | . | . | . |
| Placebo:Apixabban | 2 | 0.44 | 0.72 | 0.59 | 0.83 | 0.71 | -1.27 | 0.2036 |
| Rivaroxaban:hpixaban | 0 | 0 | 0.87 | . | 0.87 | . | . | . |
| Warfarin:hpixaban | 5 | 0.89 | 0.91 | 0.95 | 0.68 | 1.40 | 1.27 | 0.2036 |
| Dabigatran:Placebo | 0 | 0 | 1.51 | . | 1.51 | . | . | . |
| Dabigatran:Rivaroxaban | 0 | 0 | 1.25 | . | 1.25 | . | . | . |
| Dabigatran:farfarin | 1 | 1.00 | 1.19 | 1.19 | . | . | . | . |
| Placebo:Rivaroxaban | 0 | 0 | 0.82 | . | 0.82 | . | . | . |
| Placebo:Narfarin | 2 | 0.67 | 0.78 | 0.87 | 0.62 | 1.40 | 1.27 | 0.2036 |
| Rivaroxaban:Warfarin | 4 | 1.00 | 0.95 | 0.95 | . | . | . | . |

**Note:** K: Number of studies providing direct evidence, prop: Direct evidence proportion, nma: Estimated treatment effect (HR) in network meta-analysis, direct: Estimated treatment effect (HR) derived from direct evidence, indirect: Estimated treatment effect (HR) derived from indirect evidence, RoR: Ratio of Ratios (direct versus indirect), z: z-value of test for disagreement (direct versus indirect), P-value: P-value of test for disagreement (direct versus indirect).

**Supplementary Table 8** Inconsistency test of intracranial bleeding

| **Comparison** | **k** | **prop** | **nma** | **direct** | **indirect** | **ROR** | **z** | **P-value** |
| --- | --- | --- | --- | --- | --- | --- | --- | --- |
| Rivaroxaban:Apixaban | 0 | 0 | 0.90 | . | 0.90 | . | . | . |
| Warfarin:Apixaban | 1 | 1.00 | 1.11 | 1.11 | . | . | . | . |
| Rivaroxaban:Warfarin | 2 | 1.00 | 0.81 | 0.81 | . | . | . | . |

**Note:** K: Number of studies providing direct evidence, prop: Direct evidence proportion, nma: Estimated treatment effect (HR) in network meta-analysis, direct: Estimated treatment effect (HR) derived from direct evidence, indirect: Estimated treatment effect (HR) derived from indirect evidence, RoR: Ratio of Ratios (direct versus indirect), z: z-value of test for disagreement (direct versus indirect), P-value: P-value of test for disagreement (direct versus indirect).

**Supplementary Table 9** Inconsistency test of minor bleeding

| **Comparison** | **k** | **prop** | **nma** | **direct** | **indirect** | **ROR** | **z** | **P-value** |
| --- | --- | --- | --- | --- | --- | --- | --- | --- |
| Rivaroxaban:Dabigatran | 0 | 0 | 1.06 | . | 1.06 | . | . | . |
| Warfarin:Dabigatran | 1 | 1.00 | 0.93 | 0.93 | . | . | . | . |
| Rivaroxaban:narfarin | 2 | 1.00 | 1.13 | 1.13 | . | . | . | . |

**Note:** K: Number of studies providing direct evidence, prop: Direct evidence proportion, nma: Estimated treatment effect (HR) in network meta-analysis, direct: Estimated treatment effect (HR) derived from direct evidence, indirect: Estimated treatment effect (HR) derived from indirect evidence, RoR: Ratio of Ratios (direct versus indirect), z: z-value of test for disagreement (direct versus indirect), P-value: P-value of test for disagreement (direct versus indirect).

**Supplementary Figure 1** Funnel plot of mortality

**
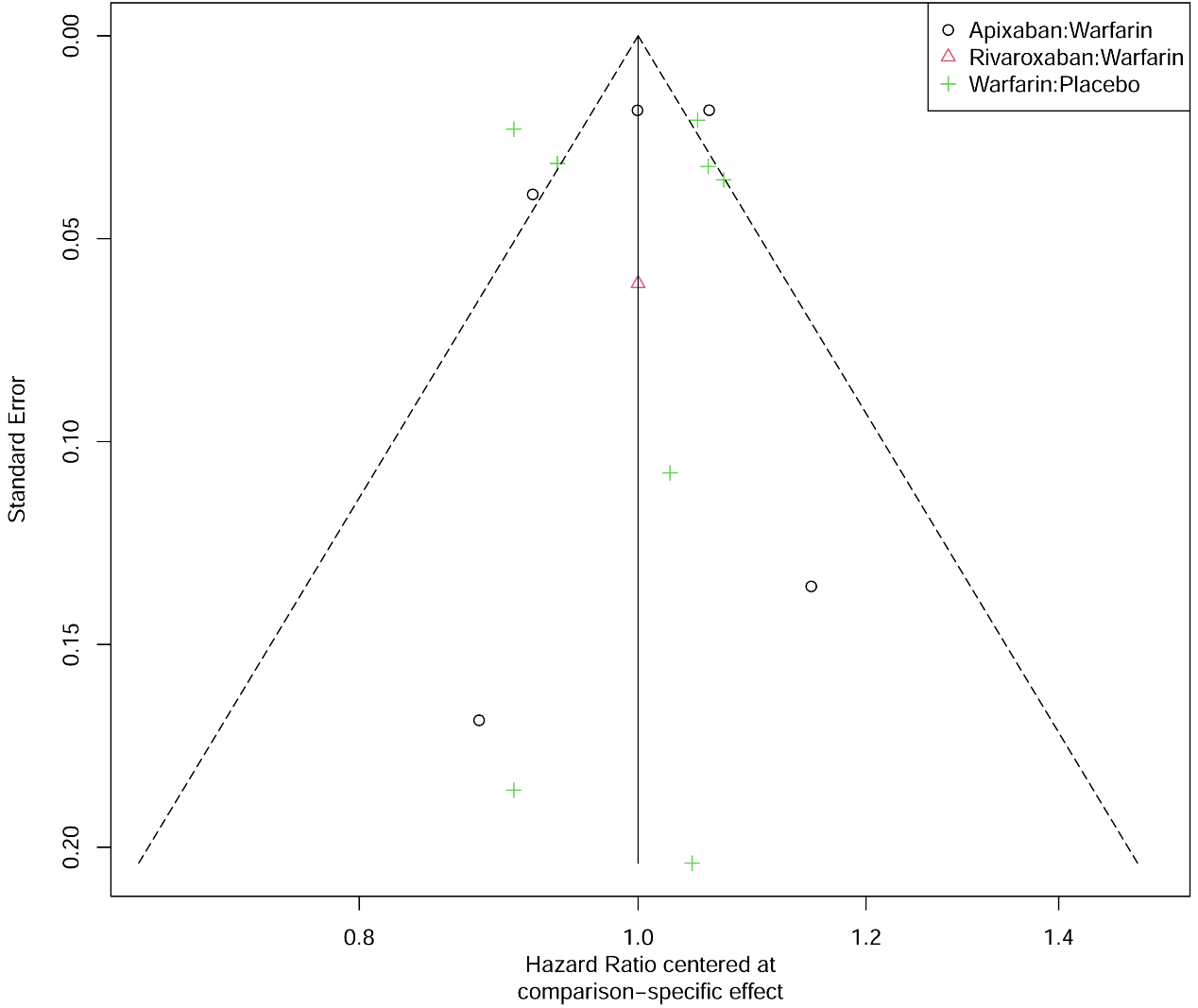
**

**Supplementary Figure 2** Funnel plot of ischemic stroke


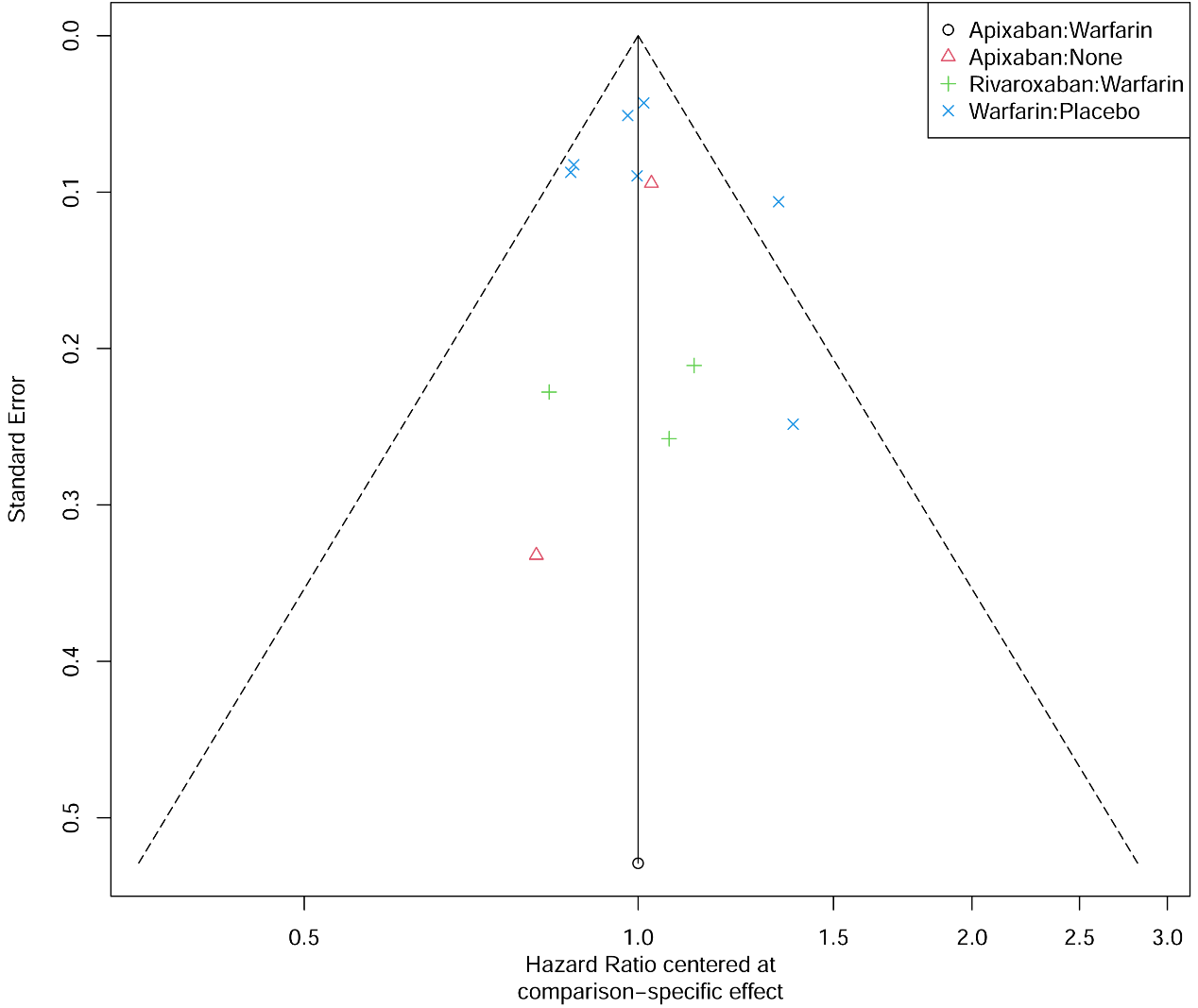


**Supplementary Figure 3** Funnel plot of hemorrhagic stroke


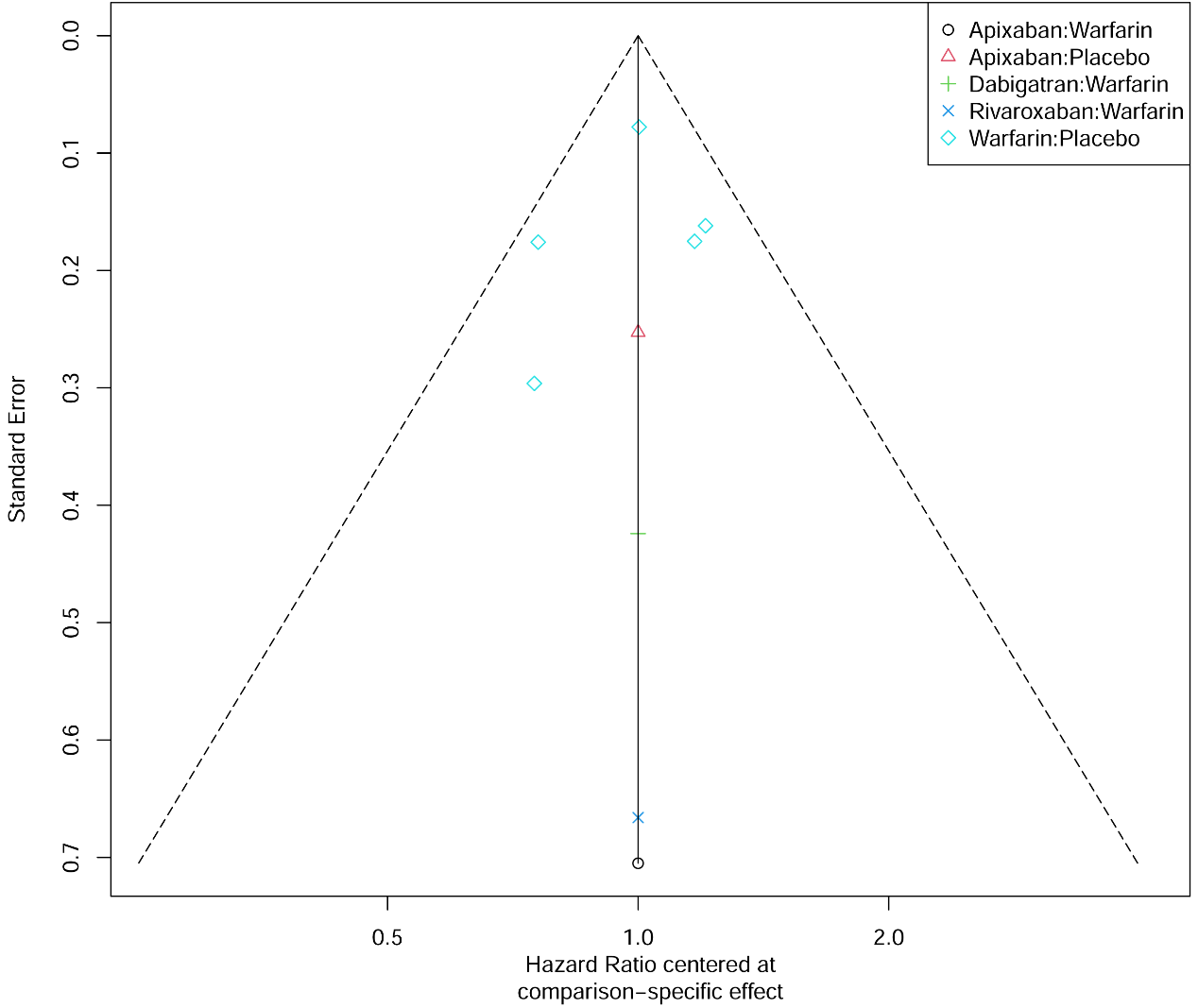


**Supplementary Figure 4** Funnel plot of any stroke


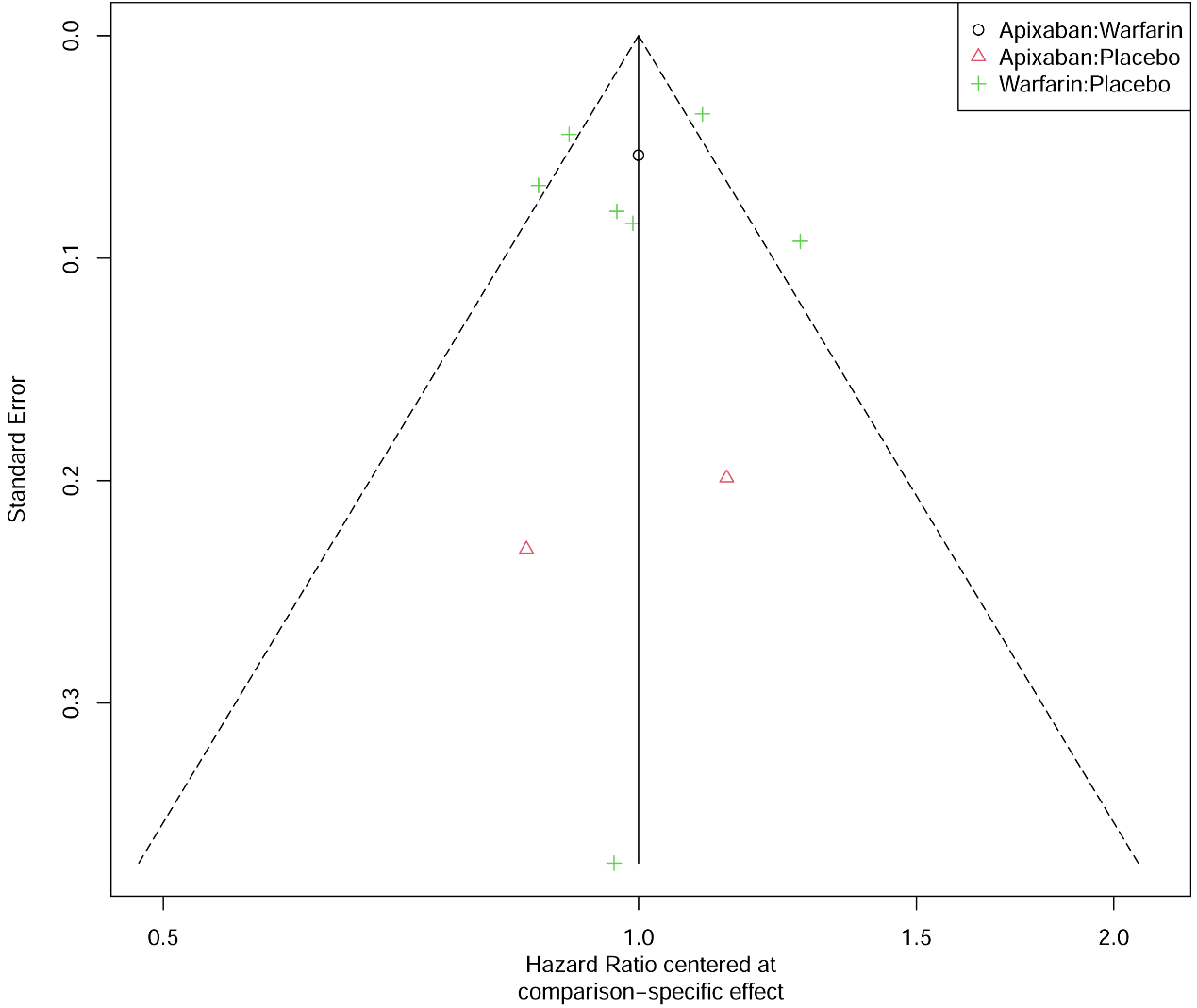


**Supplementary Figure 5** Funnel plot of gastrointestinal hemorrhage


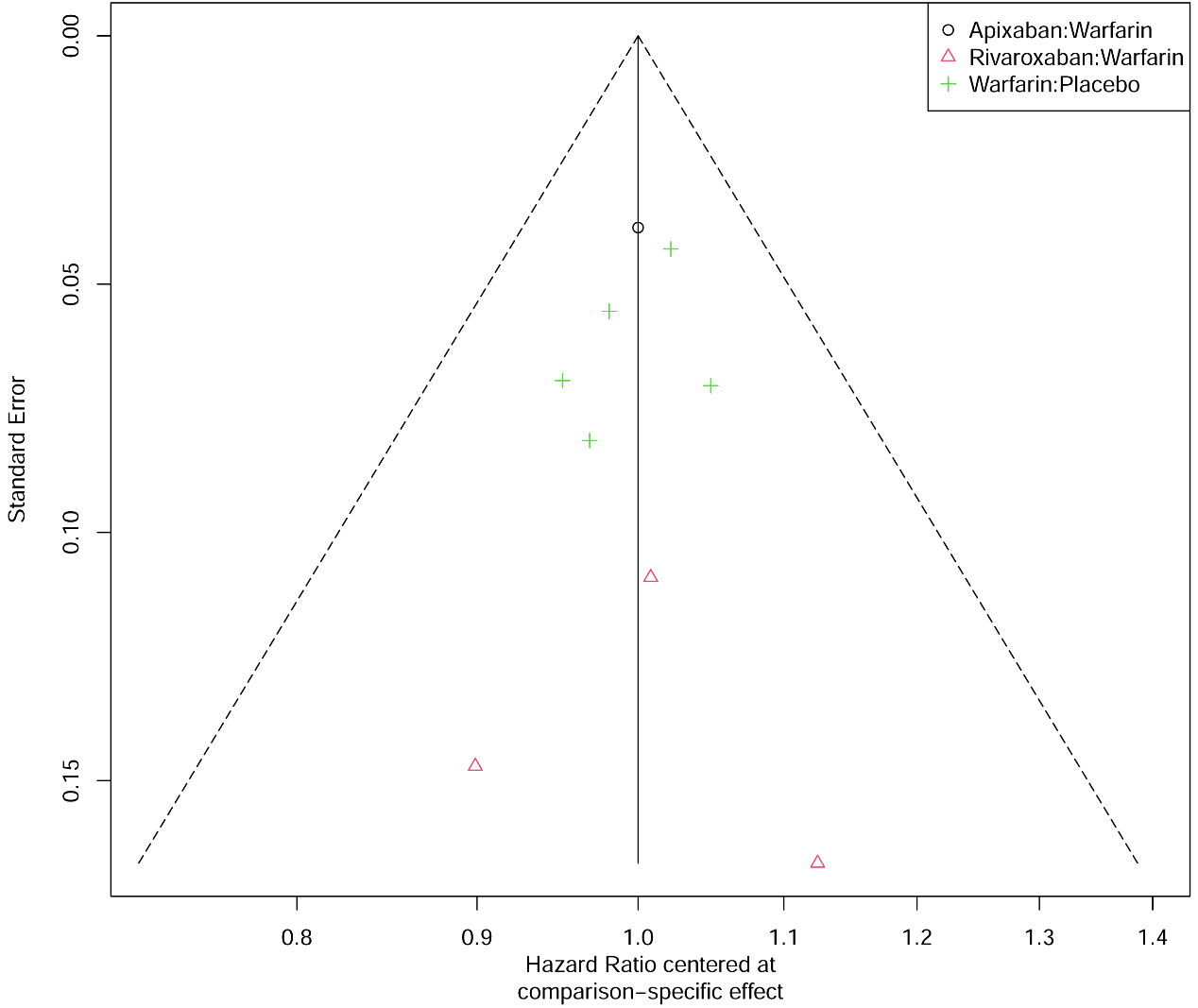


**Supplementary Figure 6** Funnel plot of major bleeding


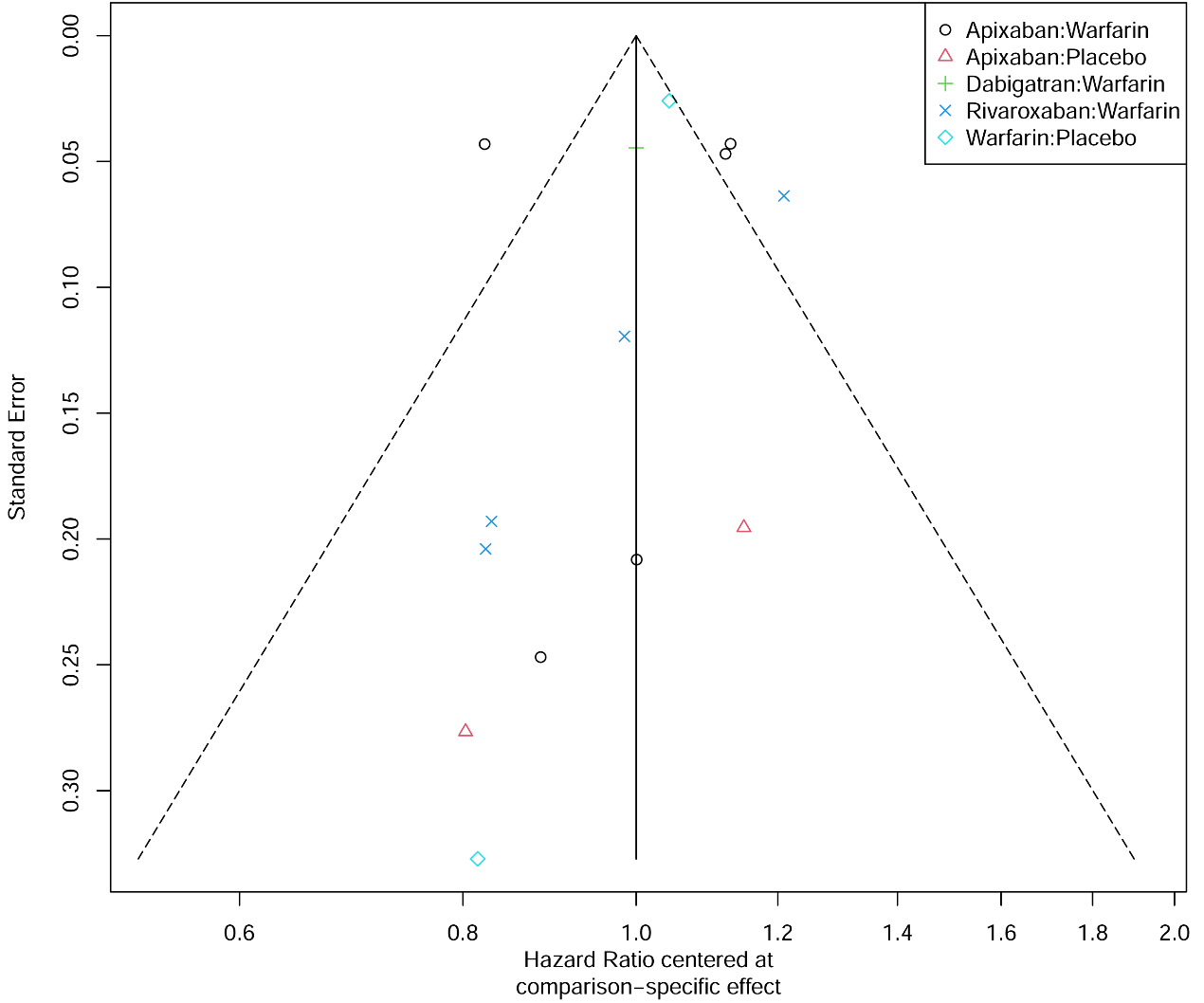


**Supplementary Figure 7** Funnel plot of intracranial bleeding


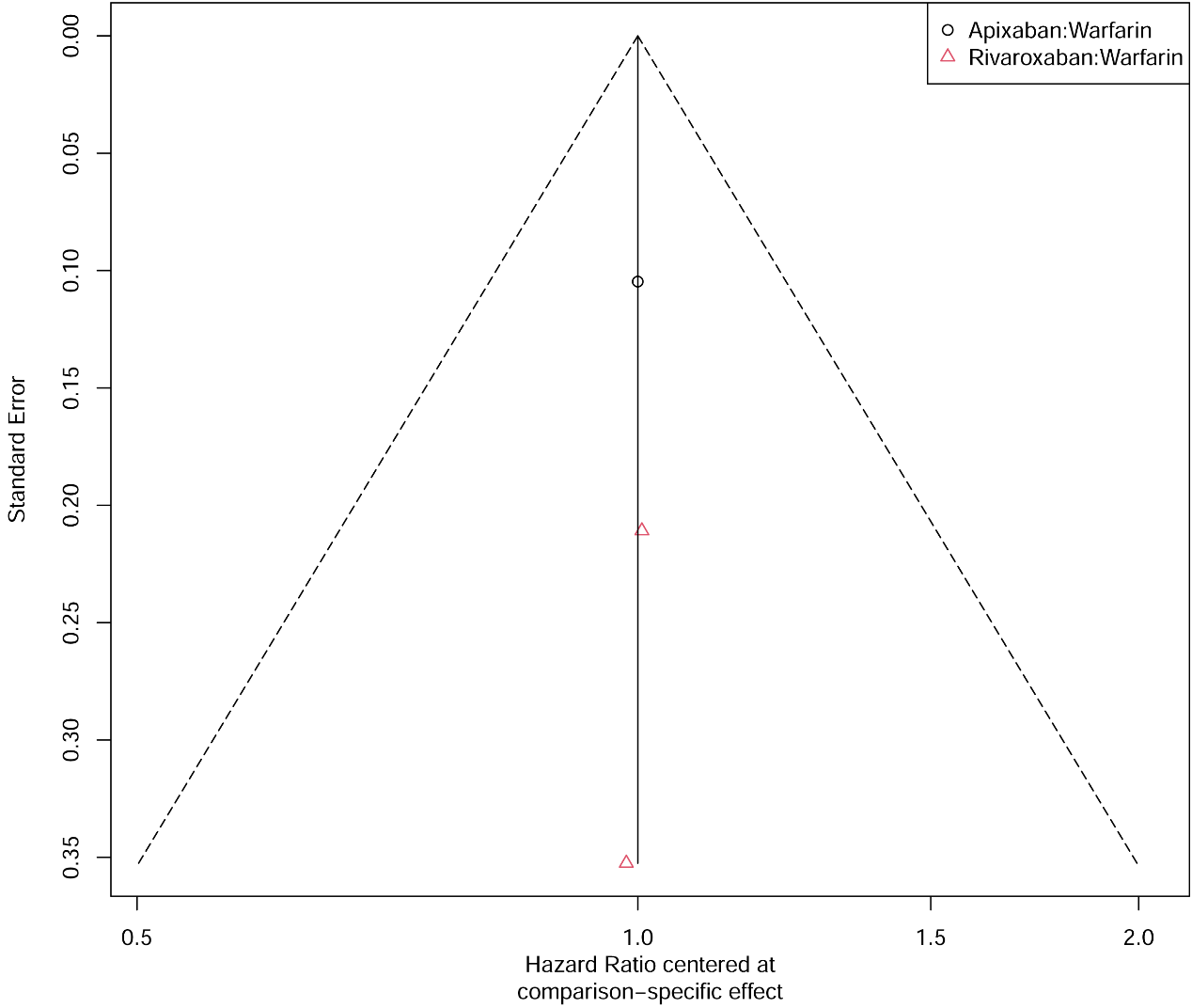


**Supplementary Figure 8** Funnel plot of minor bleeding


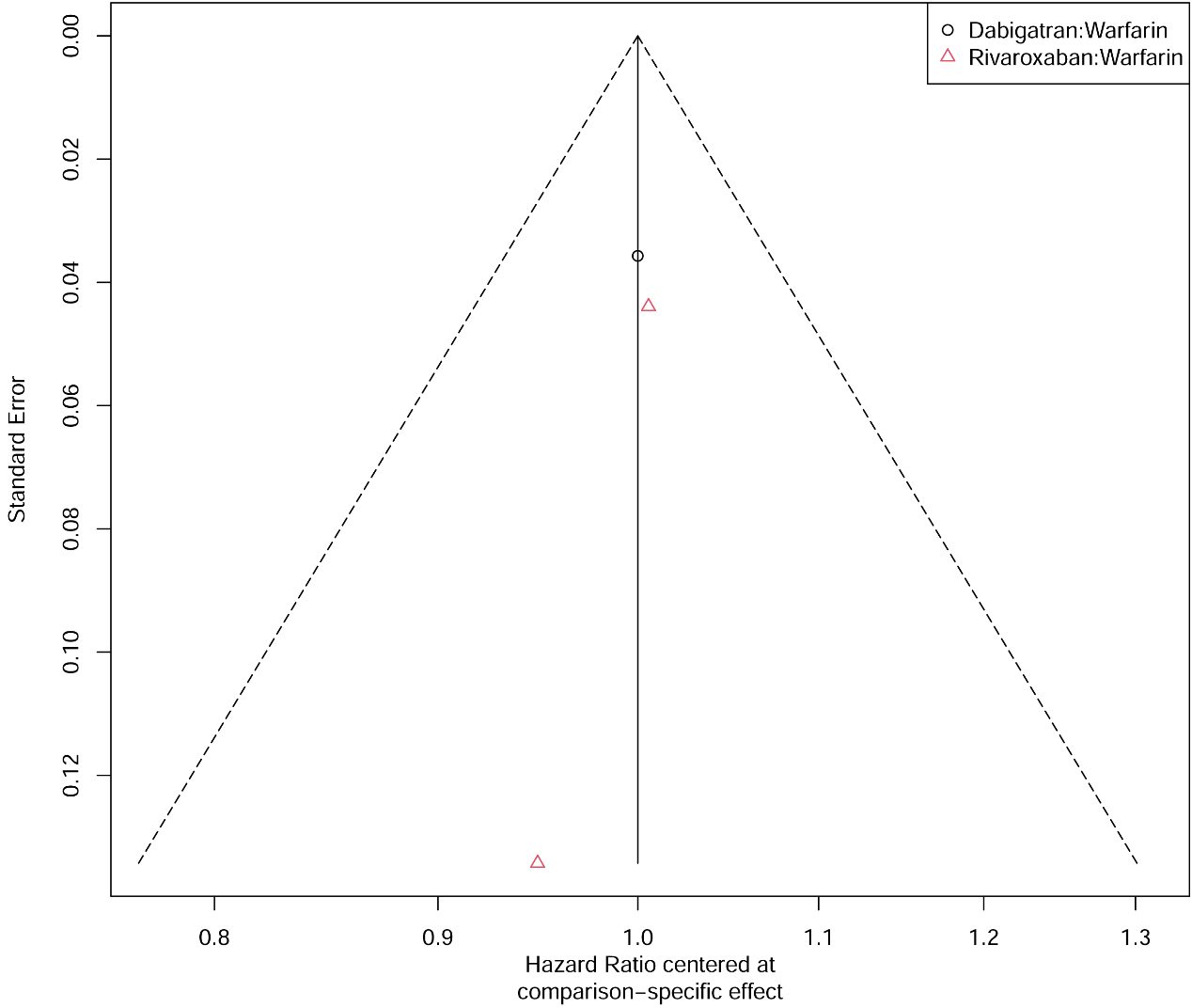

Supplement: Supplementary file 1 [file DataSheet1.docx]
